# (*E*)-2-Benzylidenecyclanones: Part XIX. Reaction of (*E*)-2-(4′-X-Benzylidene)-1-tetralones with Cellular Thiols: Comparison of Thiol Reactivities of Open-Chain Chalcones and Their Six- and Seven-Membered Cyclic Analogs

**DOI:** 10.3390/ijms25147773

**Published:** 2024-07-16

**Authors:** Fatemeh Kenari, Zoltán Pintér, Szilárd Molnár, Igor D. Borges, Ademir J. Camargo, Hamilton B. Napolitano, Pál Perjési

**Affiliations:** 1 Institute of Pharmaceutical Chemistry, University of Pécs, H-7624 Pécs, Hungary; kenari.fatemeh@gmail.com (F.K.); pinter.zoltan@pte.hu (Z.P.); molnar.szilard@pte.hu (S.M.); 2 Research Institute for Viticulture and Oenology, University of Pécs, H-7634 Pécs, Hungary; 3 Grupo de Química Teórica e Estrutural de Anápolis, Universidade Estadual de Goiás, Anápolis 75132-903, GO, Brazil; i.dalarmelino@gmail.com (I.D.B.); ajc@ueg.br (A.J.C.); hamilton@ueg.br (H.B.N.)

**Keywords:** chalcone, glutathione, cysteine, thiols, Michael addition, diastereoselective addition

## Abstract

Non-enzyme-catalyzed thiol addition onto the α,β-unsaturated carbonyl system is associated with several biological effects. Kinetics and diastereoselectivity of non-enzyme catalyzed nucleophilic addition of reduced glutathione (GSH) and N-acetylcysteine (NAC) to the six-membered cyclic chalcone analogs **2a** and **2b** were investigated at different pH values (pH 3.2, 7.4 and 8.0). The selected compounds displayed in vitro cancer cell cytotoxicity (IC_50_) of different orders of magnitude. The chalcones intrinsically reacted with both thiols under all incubation conditions. The initial rates and compositions of the final mixtures depended both on the substitution and the pH. The stereochemical outcome of the reactions was evaluated using high-pressure liquid chromatography with UV detection (HPLC-UV). The structures of the formed thiol-conjugates and the retro-Michael products (*Z*)-**2a** and (*Z*)-**2b** were confirmed by high-pressure liquid chromatography-mass spectrometry (HPLC-MS). Frontier molecular orbitals and the Fukui function calculations were carried out to investigate their effects on the six-membered cyclic analogs. Data were compared with those obtained with the open-chain (**1**) and the seven-membered (**3**) analogs. The observed reactivities do not directly relate to the difference in in vitro cancer cell cytotoxicity of the compounds.

## 1. Introduction

Chalcones (**1**) are open-chain intermediary compounds of the biosynthetic pathway of flavonoids, the largest class of low-molecular-weight polyphenolic secondary metabolites [1,2]. Among the naturally occurring chalcones and their synthetic analogs, several compounds display antibacterial, antifungal, antiviral, antimalarial, antituberculous and antiparasitic properties. Several chalcones are also effective as cell-proliferating inhibitors, antitumor-promoting, anti-inflammatory and chemopreventive agents [3,4,5,6,7,8,9,10,11].

The two main types of molecular mechanisms of the compounds are their (a) non-covalent interactions with biological macromolecules and (b) covalent modification of preferably the soft nucleophilic thiol function(s) of amino acids, peptides and proteins [7,8,9,10,11,12,13]. This latter reaction can alter the intracellular redox status, which can modulate events such as deoxyribonucleic acid (DNA) synthesis, enzyme activation, selective gene expression and cell cycle regulation [14,15]. Several biological effects (e.g., nuclear factor κB (NF-κB) pathway inhibition (anti-inflammatory effect) [16,17], activation of the nuclear factor erythroid 2-like 2 (Nrf2) pathway (antitumor/cytoprotective effect) [17,18,19], inhibition of protein kinases (antitumor effect) [7,8,9] and interaction with tubulin at colchicine binding site (antimitotic effect) [6,7,8,9,10,11,12]) of chalcones have been associated with their Michael-type reactivity toward cysteine residues of proteins. It has been suggested that the lower glutathione-depletion potential of chalcones with strong electron donor substituents (e.g., dimethylamino) on the B ring could be the consequence of the lower Michael-type reactivity of the derivatives toward GSH [20]. On the other hand, higher reactivity toward GSH and other thiols has been found to be parallel with higher NAD(P)H quinone dehydrogenase 1 (NQO1)-inducing potential of the investigated chalcones [21].

**Figure 1:**
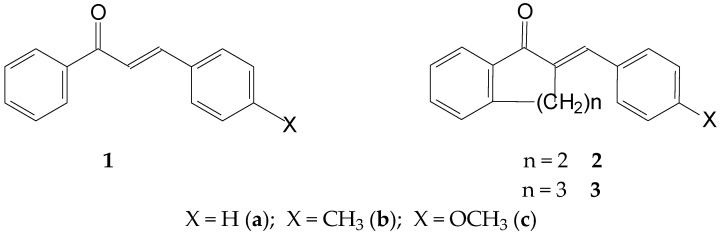
Structural formula and numbering of 4-X-chalcones (**1**) and (E)-2-(4′-X-phenylmethylene)-1-tetralones (**2**) and -benzosuberones (**3**).

The chalcone (**1**) structure comprises three structural units: the aromatic rings A and B and the propenone linker (Figure 1). The three structural elements form a conjugated system. Modifying the structure of the structural units can change both the planarity (extension of conjugation) and the reactivity of the molecules. Accordingly, the proper selection of the substituents can direct the main feature of interactions of the synthetic derivatives towards the non-covalent or the covalent pathway. Our previous studies investigated how the nature of the B-ring substituent and the ring size (n = 5–7) of cyclic chalcone analogs affect the cancer cell cytotoxic effect of more than 100 derivatives. It was found that the relative position of the two aromatic rings, as well as the electronic properties of the aromatic substituents, plays a determining role in the cancer cell toxicity of the compounds [22,23,24]. Selected IC_50_ data from the results are shown in Table 1.

In our earlier works, the reactivity of the open-chain **1b**, **1c** and their seven-membered cyclic analogs **3b** and **3c** with GSH and NAC was investigated under three conditions with different pH: (a) pH 8.0, (b) pH 6.3 and (c) pH 3.2 [1,25]. It was found that both open-chain chalcones (**1b** and **1c**) showed high and comparable reactivity under each condition [25]. On the contrary, between the cyclic analogs, the less cytotoxic methyl substituted **3b** showed a higher reactivity [1]. In the comparison of the cytotoxicities of respective six- (**2b** and **2c**) and seven-membered (**3b** and **3c**) derivatives, a reduction in cytotoxicities was observed in both pairs (Table 1).

The present work investigated how the ring size and the 4′-substitution affect the thiol-reactivity of **2b** and **2c**. A comparison of the initial reactivities of the open-chain **1a** and **1b** with the respective **3a** and **3b** showed that the incorporation of a seven-membered ring into the chalcone moiety reduced the initial reactivity of the cyclic analogs towards the thiol nucleophiles [1]. However, no previous data have been published on the effect of the ring size of cyclic chalcone analogs on their reactivity towards thiols. The diastereomeric selectivity of the addition reactions could also be investigated using the earlier HPLC-UV method [1].

**Figure 2:**
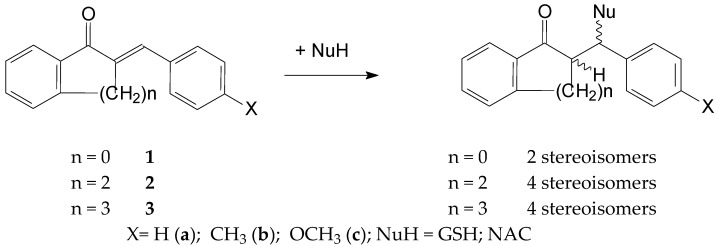
A simplified procedure for the addition of GSH and NAC to chalcones (**1**) and cyclic chalcone an-alogs (**2,3**).

Thiol additions to the electrophilic beta-carbon atom of enones (thia-Michael reactions) are reported to be a reversible process. The base-catalyzed thiol addition involves three consecutive steps: (a) deprotonation of the thiol, (b) addition of the formed thiolate to give an enolate intermediate and (c) protonation of the enolate to give the neutral adduct [25]. Due to the inherent chirality of GSH and NAC, the addition of these thiols to chalcones (1) results in the formation of two diastereomeric adducts. A similar reaction of the cyclic chalcone analogs (**2** and **3**) gives four isomeric adducts (Figure 2).

To qualitatively characterize the progress of the reactions, the composition of the incubation mixtures was analyzed at the 15, 45, 75, 105, 135, 165, 195, 225, 255, 285 and 315 min timepoints by HPLC-UV. Furthermore, density functional theory (DFT) calculations were used to analyze the stability and regioselectivity of chalcone analogs on a structural basis. The analyses used methanethiol (**CH_3_SH**) and its deprotonated form (**CH_3_S^−^**) as model thiols.

## 2. Results

### 2.1. Reactions under Slightly Basic (pH 8.0) Conditions

**Figure 3:**
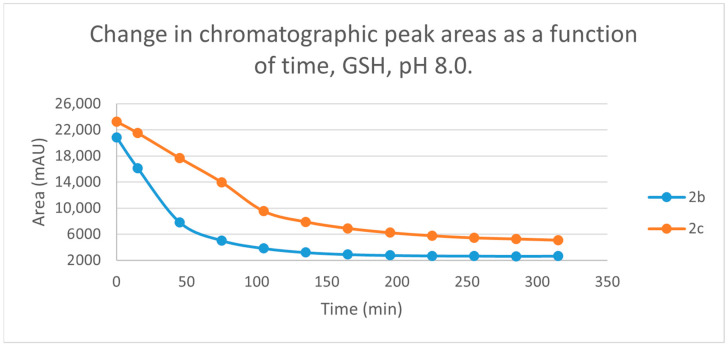
Change in the chromatographic peak area of chalcones **2b** and **2c** in the chalcone-GSH incubations at pH 8.0.

**Figure 4:**
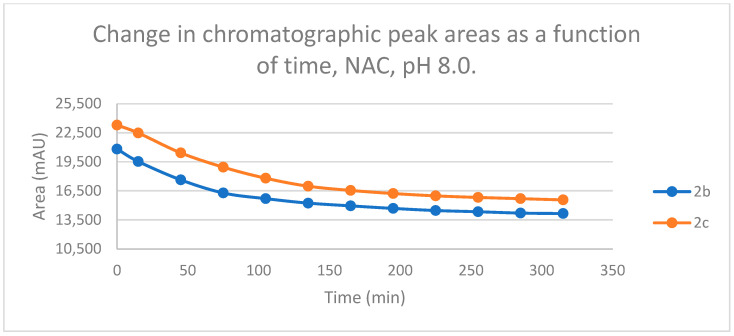
Change in the chromatographic peak area of chalcones **2b** and **2c** in the chalcone–NAC incubations at pH 8.0.

Initially, we investigated the reactions of **2b** and **2c** under basic conditions. The basic pH was selected because such conditions mimic that of the GST-catalyzed reactions, in which the ionization of the GSH thiol-function is increased due to its interaction with the basic imidazole N-atom in the enzyme’s active site [26,27]. Considering the p*K*a values of GSH (p*K*_a_ 8.83) and NAC (p*K*_a_ 9.52) [28], 12.8% of the GSH and 2.9% of the NAC molecules were under pH 8.0 conditions. Under such conditions, both GSH (Figure 3) and NAC (Figure 4) showed intrinsic reactivity with the investigated cyclic chalcone analogs.

By the end of the incubation period (315 min) with GSH, the initial HPLC-UV area of the parent compounds **2b** and **2c** was reduced by 87.2% and 78.1%, respectively (Table 2). While the compounds were incubated with NAC, the respective figures were 32.0% and 33.2% (Table 3), showing the higher reactivity of the GSH thiolate. Changes in the chromatographic peak areas of the starting chalcones as a function of the incubation time indicated that the compositions reflect an equilibrium both in the GSH and the NAC incubations. 

As a result of the addition reactions, two new chiral centers were formed. Considering the inherent chirality of the two thiols, the formation of four diastereomeric adducts was expected. In the GSH-incubation of **2c**, two separate peaks could be detected under the present chromatographic conditions. On the contrary, HPLC analysis of the **2b**/GSH incubates showed only one chromatographic peak. An analysis of the 315 min sample of the **2c**/GSH incubate showed about twofold excess of the more polar (GSH-1) diastereomers (Table 2). Contrary to the GSH-incubations, an analysis of the NAC-incubations showed two separated chromatographic peaks for both compounds (Table 3). The structure of the GSH (Figures S7–S11) and NAC conjugates (Figures S12–S17) were verified by HPLC-MS.

**Figure 5:**
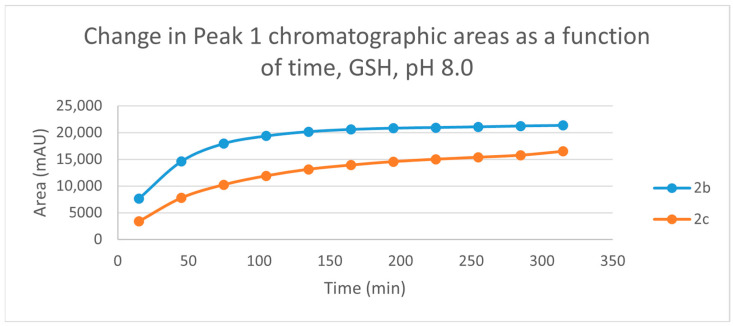
Change in the chromatographic peak area of adduct 1 of **2b** and **2c** in the chalcone-GSH incubations at pH 8.0.

**Figure 6:**
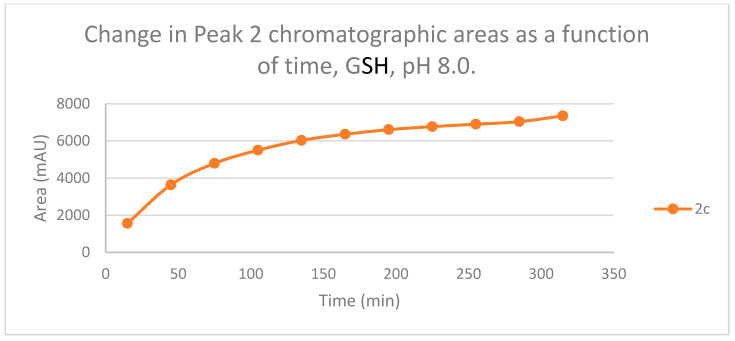
Change in the chromatographic peak area of adduct 2 of **2c** in the chalcone-GSH incubationsat pH 8.0.

**Figure 7:**
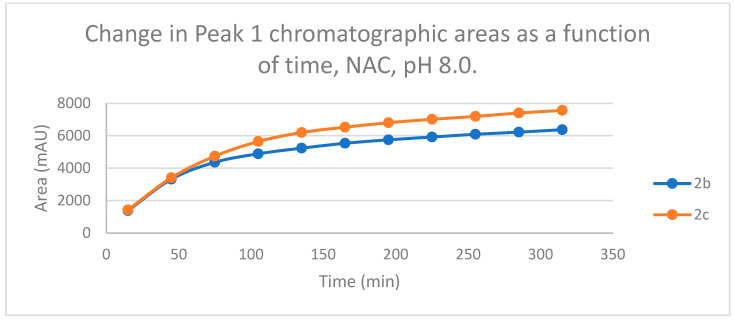
Change in the chromatographic peak area of adduct 1 of **2b** and **2c** in the chalcone-NAC incubations at pH 8.0.

**Figure 8:**
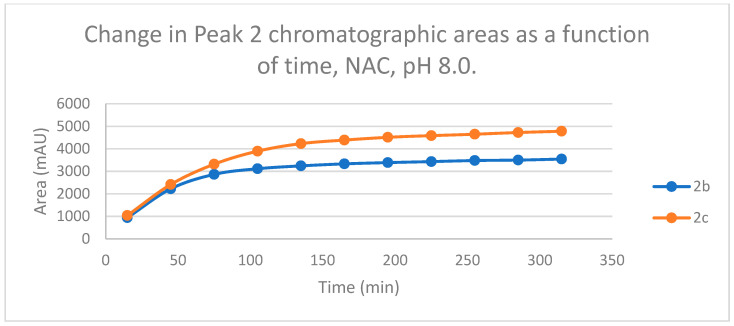
Change in the chromatographic peak area of adduct 2 of **2b** and **2c** in the chalcone-NAC incubations at pH 8.0.

The time course of the adducts’ peak increase has a concave shape in each case (Figure 5, Figure 6, Figure 7 and Figure 8). The initial curvature of the concave curves of the NAC incubates is similar at the beginning but differs from the 75-min timepoint (Figure 7 and Figure 8). The time course of increase of the more polar diastereomeric adducts (GSH-1) of **2b** and **2c** differs from the beginning. Over the incubation period, the ratio of the GSH-1/GSH-2 peak areas of **2c** was about 2 (between 2.1 and 2.2) (Figure 5 and Figure 6). The similar ratios of the NAC-1/NAC-2 peak areas of **2b** and **2c** were about 1.5, between 1.5 and 1.8 and 1.4 and 1.6, respectively (Figure 7 and Figure 8). The formation of the (*Z*)-isomer of the initial **2b** and **2c** could only be detected in the GSH-incubation of **2c** in very small (negligible) amounts (Table 2 and Table 3).

### 2.2. Reactions under Slightly Acidic (pH 6.3) Conditions

**Figure 9:**
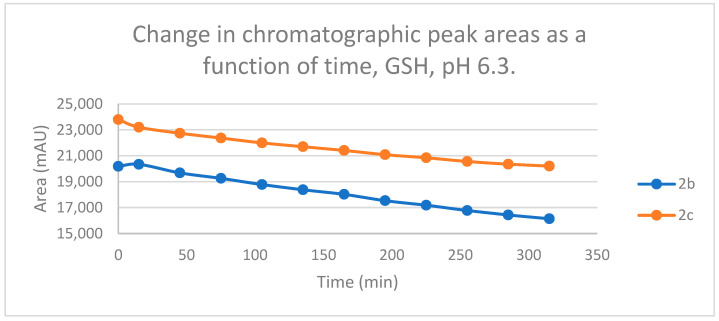
Change in the chromatographic peak area of chalcones **2b** and **2c** in the chalcone–GSH incubations at pH 6.3.

**Figure 10:**
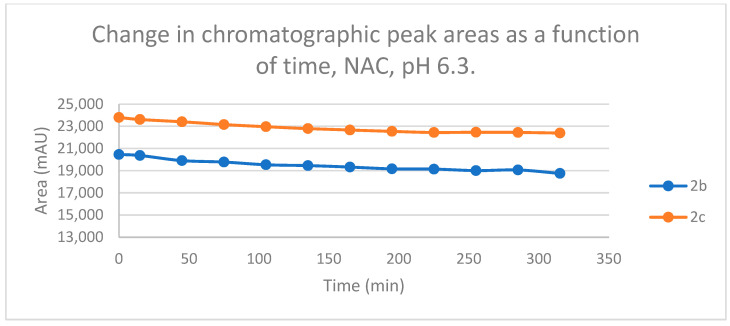
Change in the chromatographic peak area of chalcones **2b** and **2c** in the chalcone–NAC incubations at pH 6.3.

Reactions under slightly acidic conditions mimic the cellular milieu of the cancer cells [29]. Under such conditions, about 0.3% of the GSH molecules and 0.06% of the NAC molecules exist in the more reactive thiolate form. According to the expectations, the progress of the reactions under such conditions is more restricted than that observed at pH 8.0. The change in the chromatographic peak areas (concentrations) of the starting chalcones **2b** and **2c** showed parallelism in both reactions (Figure 9 and Figure 10). By the end of the incubation period (315 min) with GSH, the initial area of the HPLC peak of **2b** and **2c** was reduced by 20.1% and 15.1%, respectively. While the compounds were incubated with NAC, the respective figures were 8.3% and 5.9%. These figures were much lower than those obtained under slightly basic conditions (Table 2 and Table 3).

**Figure 11:**
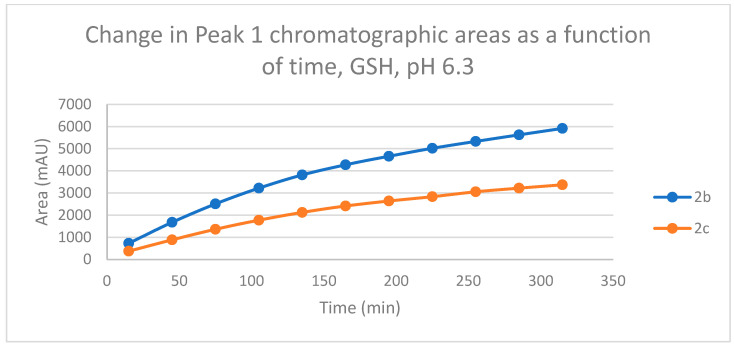
Change in the chromatographic peak area of adduct 1 of **2b** and **2c** in the chalcone-GSH incubations at pH 6.3.

**Figure 12:**
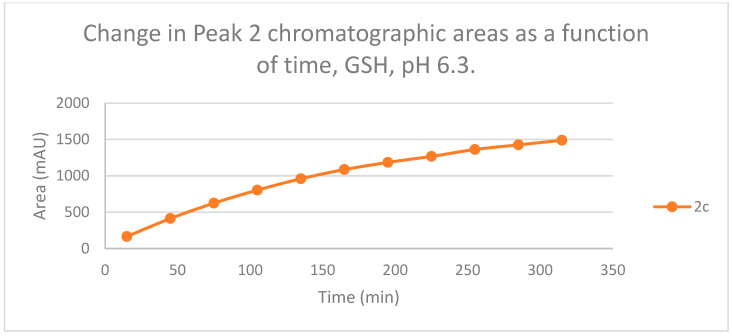
Change in the chromatographic peak area of adduct 2 of **2c** in the chalcone-GSH incubations at pH 6.3.

The HPLC chromatograms of the thiol-adducts of the two cyclic chalcone analogs showed differences. In the GSH incubation of **2c**, two separate peaks could be detected. On the other hand, HPLC analysis of the **2b**/GSH incubates—similar to the results obtained in the pH 8.0 incubations—showed only one chromatographic peak. The GSH-1 peak areas of the **2b** and **2c** increased closely parallelly over time (Figure 11). Over the whole incubation period, the ratio of the GSH-1/GSH-2 diastereomeric peak areas of **2c**—similar to the results obtained in the pH 8.0 incubations—was about 2 (between 2.2 and 2.3) (Figure 11 and Figure 12).

HPLC chromatograms of the chalcone-NAC adducts showed a different pattern. In this case, HPLC analysis of the **2b**/NAC incubates showed two small, separated peaks. On the contrary, in the chromatograms of the **2c**/NAC incubates, only one peak appeared (Figures S18 and S19). It is worth mentioning that the peak area of the more polar adducts was found in excess in both the GSH-1/GSH-2 ratio of **2c** and the NAC-1/NAC-2 ratio of **2b**. HPLC-UV analysis showed the presence of the (*Z*) isomer only in the case of the **2c**/GSH and the **2c**/NAC incubations in very small amounts (Table 2 and Table 3).

### 2.3. Reactions under Acidic (pH 3.2) Conditions

**Figure 13:**
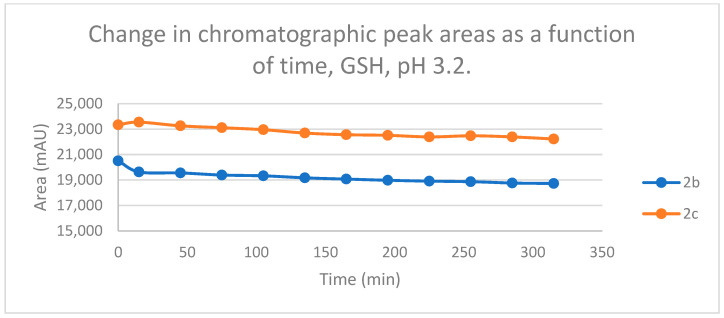
Change in the chromatographic peak area of chalcones **2b** and **2c** in the chalcone–GSH incubations at pH 3.2.

Under such strongly acidic conditions, the thiol function of GSH and NAC exists exclusively in neutral (protonated) form. According to the lower reactivity of the neutral nucleophiles [30], the reduction in the initial area of **2b** and **2c** in the chalcone-GSH incubations showed very slight downhill linear curves (Figure 13). By the end of the incubation period (315 min), the initial area of the starting **2b** and **2c** was reduced by 8.7% and 4.8%, respectively (Table 2 and Table 3).

In parallel, a linear increase of two **2c**-GSH adducts (GSH-1 and GSH-2) was observed. In the case of **2b**, only one chromatographic peak could be detected (Figures S20 and S21). Over the 315 min incubation period, the ratio of the GSH-1/GSH-2 diastereomeric peak areas of **2c** was about 3 (between 3.2 and 3.3) (Figures S20 and S21). The formation of the (*Z*)-isomer of the initial chalcones could only be detected in the GSH-incubation of **2c**. However, the peak area of the (Z)-**2c** was also very low under these conditions (Table 2 and Table 3).

In the chalcone-NAC incubations, the reduction in the initial area of the chalcones also showed a relatively slight downhill linear shape (Figure S22). By the end of the incubation period (315 min), the initial peak area of **2b** and **2c** was reduced by 30.8% and 23.6%, respectively (Table 3). Similar to the results obtained in the pH 6.3 incubations, only one **2c-NAC** adduct could be detected in the HPLC-UV chromatograms (Figure S24). The ratio of the **2b-NAC** isomeric peaks continuously increased (from 0.74 to 1.79) and reached its maximum (1.79) at the 315-min timepoint (Figures S23 and S24). Besides the identified compounds, several other small peaks appeared in the chromatograms. Similar results were observed in the NAC-incubation of the seven-membered analogs **3b** and **3c** [1]. The structures of the formed products could not be identified.

### 2.4. Molecular Modeling Analysis

**Figure 14:**
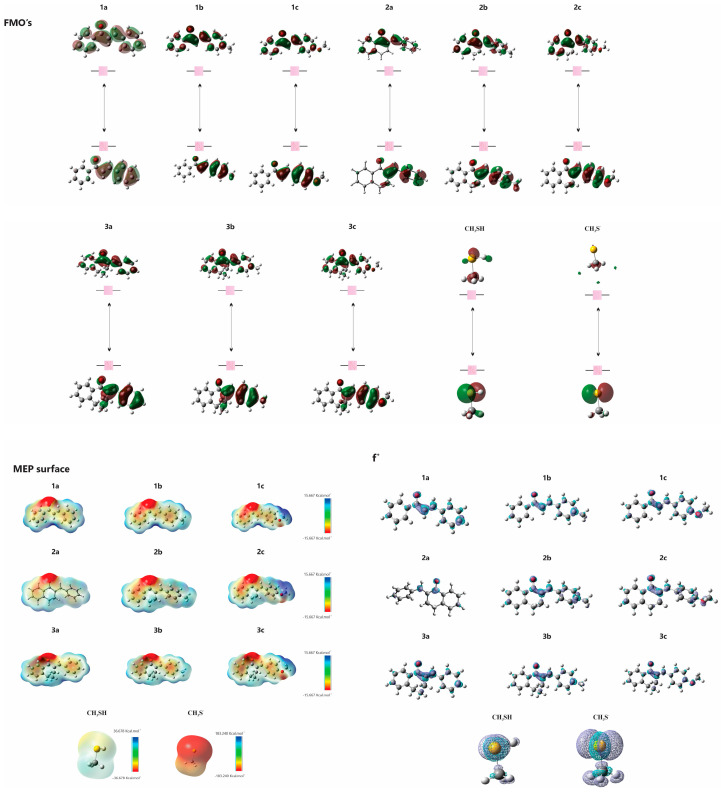
HOMO and LUMO plots for molecules (**1a**–**c**, **2a**–**c** and **3a**–**c**) calculated at the M06-2X/6-311++G(d,p) level of theory. MEP surface at ρ(r) = 4.0 × 10^−4^ electrons/Bohr3 contour of the total SCF electronic density for molecules. Isosurfaces of the nucleophilic attack (f^+^) for molecules at the M06-2X/6-311++G(d,p) level of theory.

The electronic structures of **1a**–**c**, **2a**–**c**, **3a**–**c**, **CH_3_SH** and **CH_3_S^−^** were studied using computational methods. The data, including those previously published for **1a** and **3a** [1], are summarized in Table 4. The analysis revealed that the energies of the highest occupied molecular orbital (*E*_HOMO_) and the lowest unoccupied molecular orbital (*E*_LUMO_) are crucial for understanding the electron-donating and accepting capabilities of the molecules, respectively. The (ΔE_LUMO-HOMO_) differences assist in comprehending chemical stability. Fukui functions suggest preferred sites for nucleophilic attacks, while molecular electrostatic potential (MEP) maps highlight regions vulnerable to electrophilic and nucleophilic attacks (Figure 14).

## 3. Discussion

**Figure 15:**
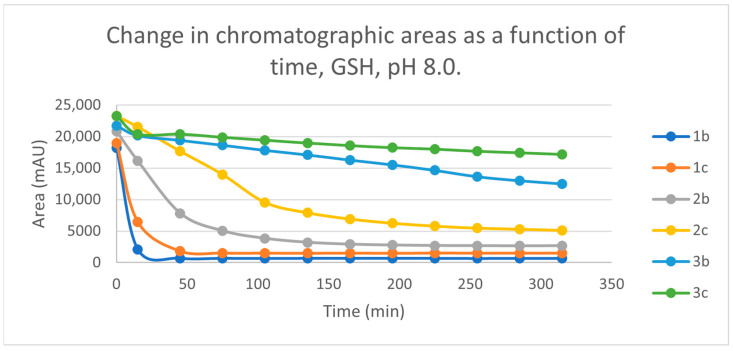
Change in the chromatographic peak area of chalcones **1b, 1c** [25]**, 2b**, **2c** and **3b, 3c** [1] in the chalcone-GSH incubations at pH 8.0.

The reactivity of chalcones and chalcone analogs with cellular thiols is considered to be one of the molecular mechanisms of their biological activity [7,8,9,10,11,12,13]. The subject of this study was the thiol reactivity of some cyclic chalcone analogs (**2b** and **2c**) that displayed different levels of in vitro cytotoxic activities toward murine and human leukemia cell lines (Table 1). Spontaneous thiol reactivity with GSH and NAC of **2b** and **2c** was investigated under the previously used in vitro conditions [1,25]. Studies performed under basic (pH 8.0) conditions—mimicking the milieu of the GST-catalyzed reactions [26,27]—showed the compounds (**2b** and **2c**) to have relatively high reactivity with GSH and NAC (Figure 3 and Figure 4). Such reactivities are comparable with those of the respective open-chain **1b** and **1c** [25]. On the other hand, both the initial reactivity and the 315 min conversion of **2b** and **2c** were much higher than those of the respective seven-membered analogs **3b** and **3c** [1]. The composition of the incubation of **2b** with GSH reached the equilibrium by the end of the 315-min incubation periods. The composition of the other GSH-chalcone incubations (pH 8.0) was also close to the equilibrium (Figure 15).

**Figure 16:**
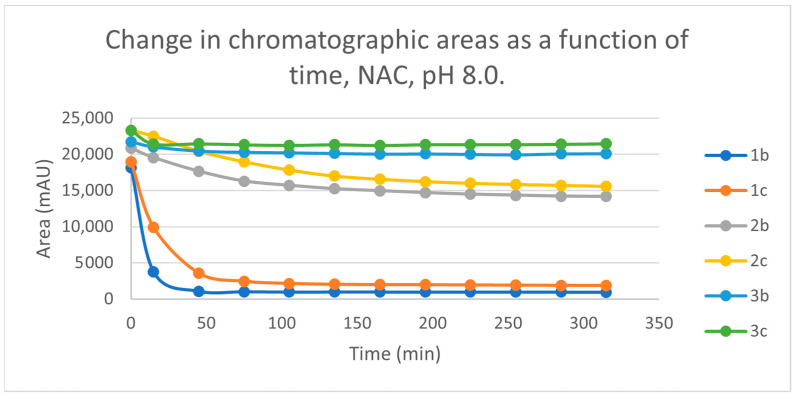
Change in the chromatographic peak area of chalcones **1b, 1c** [25]**, 2b**, **2c** and **3b, 3c** [1] in the chalcone-NAC incubations at pH 8.0.

The (quasi)equilibrium composition (pH 8.0) of the chalcones with different substituents (**b** and **c**) was similar for both GSH and NAC (Figure 15 and Figure 16). The conversion of the methyl-substituted derivatives (**b**) was somewhat higher in each case. However, the respective values of the GSH and the NAC incubations were different. The GSH incubations of chalcones **2** and **3** were much higher in the corresponding conjugates than the NAC incubations. Compositions were most shifted towards the product formation in the case of the conformationally most flexible open-chain chalcones (**1**) (Figure 15 and Figure 16). Based on these observations, the differences can be explained by the higher conformational mobilities (entropy tag) of the GSH-conjugates.

Under slightly acidic conditions (pH 6.3), the (quasi)equilibrium compositions of **2** and **3** contained much less GSH and NAC conjugates. The reactivity of the open-chain chalcones (**1b** and **1c**) was much higher than the two cyclic analogues (**2** and **3**). The reactivity of each series (**1**, **2** and **3**) was more pronounced with GSH. Similar to the pH 8.0 conditions, the conversion of the methyl-substituted (**b**) derivatives was higher in each series (Figures S25 and S26).

Under acidic (pH 3.2) conditions, the 315 min-conversions were much lower than under the above conditions. The methyl-substituted derivatives (**b**) showed somewhat higher reactivity toward both thiols. The only significant difference was the more pronounced reactivity of **1b** against GSH (Figures S27 and S28).

^13^C NMR shifts, indicating the electron density around the particular nucleus of the β-C atom of **2b** (136.8 ppm) and **2c** (136.6 ppm),—as well as that of **1b** and **1c** [25] and **3b** and **3c** [1]—were reported to be similar [31]. Accordingly, the observed difference in the reactivity of chalcones **b** and **c** can be explained by the stability of the respective thiol adducts. Humphlett et al. demonstrated that the activity of the α-hydrogen atom of the adduct, the resonance stabilization of the enone formed by cleavage and the anionic stability of the thiolate ion are the determining factors of the reverse process [32]. Since the 4′-methyl substitution can more effectively increase the electron density on the carbon-carbon double bond, and the formed chalcone is resonance-stabilized, the elimination process is more effective in the case of the 4′-OCH_3_ (**c)** than the 4′-CH_3_ (**b**) derivatives. It is also reflected in the composition of the (quasi)equilibrium mixtures of the three series: the equilibrium mixture is always reached in the respective 4′-OCH_3_ (**c**) chalcones (Figure 15 and Figure 16).

As for the isomeric composition of the thiol-adducts, in the **2b**/GSH incubations, formation of only one GSH peak was observed, deviating from the actual pH. Since the t_R_ values of the partially separated **2c-GSH** conjugates are rather close to each other, it is reasonable to presume that the formed **2b-GSH** diastereomers are not separated under the present chromatographic conditions. The diastereomeric ratio (A_(GSH-1)/_A_(GSH-2)_) of the separated **2c-GSH** conjugates showed about two times (between 2.1 and 2.2) excess of the more polar (GSH-1) peak (pH 8.0). The ratio was constant; it did not change over the incubation period (Figure 5 and Figure 6). A similar observation was obtained when the incubation of **2c** was performed under slightly acidic (pH 6.3) (Figure 11 and Figure 12) and acidic (pH 3.2) (Figures S20 and S21) conditions; the A_(GSH-1)_/A_(GSH-2)_ ratios (315 min value) were about two (2.3) and three (3.3), respectively (Table 2).

In agreement with the constant A_(GSH-1)/_A_(GSH-2)_ diastereomeric ratios, a negligible amount of **2c** (*Z*)-isomers could be detected. These observations were similar to those obtained with the respective open-chain **1b** and **1c** [25] and opposite to those obtained with the seven-membered cyclic analogs **3b** and **3b** [1]. In the latter case, much higher amounts of (*Z*)-isomers were formed under all three pH conditions. Since the incubations were kept in the dark, the retro-Michael reaction was the only source of formation of the (*Z*)-isomers.

According to the above, the diastereoselective addition of GSH onto the C=C bond of **2c** could be observed under all three experimental conditions. It can be considered that the similar reactions of **2b** are also diastereoselective; the experimental results, however, did not provide unambiguous evidence to state that. Since the highest diastereoselectivity ratio (3.3) was observed under the acidic (pH 3.2) conditions (Table 2), the results provide further experimental support to consider that the protonated thiol forms a six-membered, hydrogen-bond stabilized intermediate, of which the equatorial 4-X-phenyl group determines the structure of the adduct [33].

HPLC analysis of the NAC incubations under basic conditions (pH 8.0) showed two separated chromatographic peaks with both compounds. Over the 315 min incubation times, the ratios of the NAC-1/NAC-2 peak areas of **2b** and **2c** were between 1.5 and 1.8 and 1.4 and 1.6, respectively (Figure 7 and Figure 8). In this case, however, the change in the ratio of the two chromatographic peak areas as a function of the pH showed different patterns for the two compounds. In the case of **2c**, no NAC-2 peak could be observed under the two acidic conditions (Table 3).

On the contrary, the A_(NAC-1)/_A_(NAC-2)_ ratios of **2b** under the slightly acidic (pH 6.3) (Figures S18 and S19) and the acidic (pH 3.2) conditions (Figures S23 and S24) changed between 2.2 and 4.9 and 0.7 and 1.8, respectively. In both cases, a continuous increase in the ratios was observed over the incubation time and reached the maximum at the 315 min timepoint. Since relatively high (*Z*)-isomeric peaks were observed in the pH 6.3 incubations (Table 2), the continuously increasing A_(NAC-1)/_A_(NAC-2)_ ratio (between 2.2 and 4.9) can be partly explained by conversion of the kinetically controlled product to the thermodynamically more stable one, through a retro-Michael reaction. Investigations of the respective 4′-CH_3_ (**3b**) and 4′-OCH_3_ (**3c**) substituted seven-membered analogs also showed a similar increase in the ratio of the chromatographic areas of the NAC-conjugates accompanied by the formation of an increased amount of the respective (*Z*)-isomers [1]. However, the ratio of the NAC-1/NAC-2 areas is opposite in the two series. The numerical value of the ratio of the peaks in both series (**2** and **3**) was higher in the case of the 4′-CH_3_ derivative [1]. The retro-Michael (elimination) reaction of the thiol-adducts can result in the formation of not only the (*E*) but the (*Z*) isomers as well. Differences in the thiol-reactivity of the (*Z*) isomers can be the reason for the different levels of the isomeric adducts in the incubation mixtures.

A comprehensive evaluation of the physicochemical properties and reactivity profiles of chalcones **1a–c** and its cyclic analogs (**2a–c** and **3a–c**) with the model thiols **CH_3_SH** and **CH_3_S^−^** were undertaken. Our observations indicate that all compounds, except **CH_3_SH**, exhibit similar electron-accepting capabilities. Notably, **CH_3_SH** demonstrates greater chemical stability. An analysis of LUMO energies revealed that **1a–c** are more acidic than their cyclic counterparts **2a–c** and **3a–c**. These findings provide experimental support for enforcing the principles of the Hard and Soft Acids and Bases (HSABs) theory [30] in the investigated reactions. This theoretical framework suggests a preference for reactions between species of similar hardness, which is consistent with our observation that open-chain chalcones are more reactive under mildly basic conditions. The different reactivities of the chalcones and their cyclic analogs with the model thiols **CH_3_SH** and **CH_3_S^-^** can be interpreted by differences in conformational flexibility and steric hindrance. Our data indicate that six-membered cyclic analogs (**2a**, **2b**, **2c**) exhibit higher reactivity than their seven-membered counterparts (**3a**, **3b**, **3c**). This increased reactivity can be attributed to the greater ring strain and reduced conformational flexibility inherent to the seven-membered cyclic structures.

## 4. Materials and Methods

### 4.1. Chemicals and Reagents

L-glutathione reduced (GSH) and N-acetyl-L-cysteine (NAC) were obtained from Sigma Aldrich (Budapest, Hungary). Methanol (MeOH) CHROMASOLV gradient for HPLC was obtained from Honeywell (Honeywell, Hungary). Trifluoroacetic acid (TFA) HiperSolve CHROMANORM was obtained through VWR (Budapest, Hungary). Formic acid was obtained from Fischer Chemical. The compounds (**2a** and **2b**) were synthesized as previously described [22,23]. Their structure and (*E*)-stereochemistry were verified by ^1^H, ^13^C NMR and X-ray studies [22,23,31]. The purity and structure of the chalcone samples (**2b**, **2c**) were characterized by their melting points and TLC investigations. Authentic (*Z*)-**2b** and (*Z*)-**2c** were synthesized by light-initiated (*E*)/(*Z*) isomerization as published earlier [26]. The structure of **2a** and **2b** and their (*Z*)-isomers ((*Z*)-**2b**, (*Z*)-**2c**) were verified by HPLC-MS method (Figures S1–S6). The structure and stereochemistry of the investigated samples (**2a** and **2b**) were proved by ^1^H NMR (Figures S29 and S30). ^1^H NMR spectra were recorded on a Bruker Avance DRX 500 spectrometer (Bruker Biospin, Karlsruhe, Baden Württemberg, Germany) in CDCl_3_ solutions.

### 4.2. Preparation of Solutions

To evaluate the reactivity of the investigated chalcone analogs with thiols, reduced glutathione (GSH) and N-acetylcysteine (NAC) solutions of three different pHs—3.2, 6.3 and 8.0—were prepared. The pH of the solutions was set using 1M NaOH. The GSH and NAC solutions were prepared in water to a total volume of 1.5 cm^3^ with a concentration of 2.0 × 10^−1^ mol·L^−1^ (0.3 mmol). Chalcone solution was prepared freshly before incubation to a 4.6 volume of HPLC grade methanol (4.6 cm^3^ of 6.5 × 10^−3^ mol·L^−1^, 0.03 mmol).

The NAC or GSH solutions were mixed with the chalcone solution to a final volume of 6.1 cm^3^, to the final concentration of 4.9 × 10^−2^ mol·L^−1^ of thiol and 4.9 × 10^−3^ mol·L^−1^ of chalcone with a molar ratio of 10:1 (thiol:chalcone). The obtained solution was kept in the dark during preparation and analysis in a temperature-controlled (37 °C) water bath for 315 min. To monitor the progress of the reactions, samples were taken at timepoints 15, 45, 75, 105, 135, 165, 195, 225, 255, 285 and 315 min and analyzed by HPLC-UV.

### 4.3. HPLC-UV Measurements

The measurements were performed on an Agilent 1100 HPLC system using a UV-Vis detector. The chromatographic conditions were the same as in our previous communication [1]. Briefly, the components were separated using a Zorbax Eclipse XBD-C8 (150 mm × 4.6 mm, particle size 5 µm) column (Agilent Technologies, Waldbronn, Germany). The injection volume was 10 µL; the flow rate was 1.2 mL/min, and the column temperature was room temperature. The wavelength of detection was 260 nm. The mobile phase consisted of (A) water and 0.1% TFA and (B) methanol and 0.1% TFA. The gradient profile was as follows: an isocratic period of 40% B for 8 min followed by a linear gradient to 60% B in 4 min, continued with a second linear gradient to 90% B in 3 min and an isocratic period of 90% B for 5 min. The column was then equilibrated to its initial conditions with a 2 min linear gradient to 40%, followed by 3 min of the isocratic period.

### 4.4. HPLC-MS Measurements

The measurements were performed on HPLC Ultimate 3000 coupled with a mass spectrometer Q Exactive Focus (Dionex, Sunnyvale, CA, USA). The HPLC separation of the chalcone-GSH adducts was performed using an Accucore C18 column (150 mm × 2.1 mm, particle size 2.6 µm) and the Accucore C18 guard column (150 mm × 2.1 mm, particle size 2.6 µm). The injection volume was 5 µL; the flow rate was 0.4 mL/min. The sampler temperature was room temperature, and the column temperature was at 30 °C. The mobile phase consisted of (A) water and 0.1% formic acid and (B) methanol and 0.1% formic acid. The gradient profile was as follows: isocratic elution of 20% B for 1 min, followed by a linear gradient to 100% B in 14 min and continued with an isocratic plateau for 2 min. The column was equilibrated to 20% B in a 0.5 min linear gradient and continued isocratically for 2.5 min. Data analysis and evaluations were performed using Thermo Scientific TranceFinder version 4.1.191.0.

A Q-Exactive Focus mass spectrometer was operated with an Orbitrap mass analyzer and APCI (atmospheric pressure chemical ionization). The ionization parameters were constant during the measurement and were set to sheat gas (nitrogen gas) 30 A.U., auxiliary gas (nitrogen gas) 10 A.U. Probe heater was set to 300 °C. Capillary temperature 350 °C. The spray voltage (+) was 5000 V, and the S lens R.F. level was 50%. Spectra were acquired in the mass/charge ratio (*m*/*z*) range of 50–2000.

In the case of the chalcone-NAC adducts, the HPLC specifications were similar to the chalcone-GSH separation method except for the gradient elution timetable, which was as follows: One1 min of isocratic elution of 10% B, a linear increase to 95% B in 14 min, followed by an isocratic period of 3 min. The column was equilibrated to 10% B in 0.1 min and continued isocratically for 2.9 min. Diode array detection (DAD) was also performed at 260 nm alongside the MS analysis. Mass spectrometry specifications followed the ionization method: HESI +/− having 35,000 resolution at 200 *m*/*z* and a scan range of 100–1000 amu. The rest of the specifications were the same as the previously mentioned one.

### 4.5. Molecular Modeling Analysis

All computational analyses were performed utilizing DFT as implemented in the Gaussian16 software suite [34]. The optimization of molecular geometries was executed in the gas phase using the M06-2X hybrid functional with long-range correction in combination with the 6-311++G(d,p) basis set [35]. The frontier molecular orbital energies, specifically the highest occupied molecular orbital (HOMO) and the lowest unoccupied molecular orbital (LUMO) were calculated via DFT methods [36]. The MEP maps were constructed to represent the electrostatic potential distribution across the molecular surface visually, thus identifying regions of electron-rich and electron-deficient areas [37]. The electronic isodensity surfaces were derived from the electrostatic potentials V(**r**) [38] at point **r**, defined as

(1) $$V\left(r\right)=\sum_{\alpha }\frac{Z_{\alpha }}{\left|r_{\alpha }-r_{A}\right|}-\int \frac{\rho (r)}{r_{\alpha }-r}dr$$

where Z_α_ is the charge of nucleus α at point **r**_α_, and $\rho \left(r\right)$ is the charge density at point **r**.

The local electrophilicity indices of the molecules were determined by applying the Fukui function [39,40], which facilitated the prediction of molecular reactive sites. The Fukui function is mathematically expressed as follows:

(2) $$f\left(r\right)={\left[\frac{\partial \rho \left(r\right)}{\partial N}\right]}_{v},$$

where N is the number of electrons in the system, and the constant term v in the partial derivative is external potential.

## 5. Conclusions

The initial aim of the present study was to seek a correlation between the spontaneous reactivity of chalcones (**2b** and **2b**) with two natural thiols (GSH and NAC) and their in vitro cancer cell cytotoxicity data. As shown in Table 1, **2c** was more cytotoxic by at least one order of magnitude than **2b** in most investigated cancer cell lines. A similar result was obtained by comparing the respective cytotoxicity of **3b** and **3c** (Table 1). A comparison of the initial rates of thiol addition reactions showed the (less cytotoxic) **2b** to have higher reactivity. Furthermore, the equilibrium composition of the incubation mixtures was higher in the **2b**-thiol adducts in both cases. Similar observations could be made by comparing the respective data of **3b** and **3c** [1]. Accordingly, the observed cytotoxicity of the compounds did not show direct correlations with their thiol reactivities. On the contrary, it is reasonable to presume that the molecular basis of their cytotoxicity is related to their non-covalent interactions with cellular macromolecules [12].

In the comparison of the composition of the equilibrium mixtures, the data indicate the importance of the entropy tag of the free enthalpy change in the reactions, which is the highest in the conformative most flexible GSH-adducts of the open-chain chalcones (**1**). Stereochemical outcome of the reactions showed characteristic differences in the two thiols. In the case of GSH, the addition reactions of **2c** carried out under acid conditions (pH 3.2) showed a high diastereomeric excess (3.2–3.3) of the more polar diastereomeric pairs. Under the slightly acidic (pH 6.3) and the basic (pH 8.0) conditions, the similar excess was about 2. These findings are consistent with our earlier assumption that, as a result of the addition of the protonated (neutral) thiol group, the zwitterionic enolate forms a six-membered transition product stabilized by a hydrogen bridge; the position of the bulky phenyl group orients the stereochemistry of the resulting product [33].

## Figures and Tables

**Table 1:** IC_50_ (μM) data of selected E-2-(4′-X-benzylidene)-1-tetralones (**2**) and -benzosuberones (**3**) and the reference compound melphalan against murine (P388 and L1210) and human (Molt 4/C8 and CEM) lymphocytic leukemia cells [22].

Compound	P388	L1210	Molt 4/C8	CEM
**2a**	30.2	121 ± 104	32.4 ± 16.6	7.42 ± 0.56
**2b**	17.7	161 ± 159	>500	460 ± 69
**2c**	22.1	44.0 ± 2.9	9.41 ± 0.29	8.84 ± 0.22
**3a**	12.7	106.0 ± 31	42.7 ± 3.3	28.9 ± 0.7
**3b**	11.8	25.0 ± 7.4	21.3 ± 14.1	11.4 ± 1.6
**3c**	1.6	0.34 ± 0.02	0.47 ± 0.33	0.35 ± 0.04
Melphalan	0.22	2.13 ± 0.03	3.24 ± 0.79	2.47 ± 0.30

**Table 2:** Retention times (t_R_) ^1^ and integrated peak areas (A) of the investigated cyclic chalcone analogs (**2b** and **2c**) and their GSH adducts ^2^.

pH ^3^	Compound	t_R_ (*E*)-Isomer	Area Ratio ^4^ A_315_/A_0_	t_R_ (*Z*)-Isomer	Area (*Z*)-Isomer	t_R_ GSH-1	Area GSH-1	t_R_ GSH-2	Area GSH-2
**3.2**	**2b**	16.8	0.91	ND ^5^	-	14.7	753	ND ^5^	-
**3.2**	**2c**	16.3	0.95	16.7	51.3	13.3	565	13.5	171
**6.3**	**2b**	17.2	0.80	ND ^5^	-	15.3	5914	ND ^5^	-
**6.3**	**2c**	16.8	0.85	17.1	67.6	14.5	3371	14.6	1489
**8.0**	**2b**	17.1	0.13	ND ^5^	-	15.1	21,325	ND ^5^	-
**8.0**	**2c**	16.9	0.22	17.2	85.9	14.6	16,474	14.8	7353

^1^ Retention times in minutes; ^2^ Data refer to the average of two independent measurements at the 315 min timepoint; ^3^ pH value of the aqueous thiol solution; ^4^ ratios of peak areas measured at 0 and 315 min; ^5^ Not detectable.

**Table 3:** Retention times (t_R_) ^1^ and integrated peak areas (A) of the investigated cyclic chalcone analogs (**2b** and **2c**) and their NAC adducts ^2^.

pH ^3^	Com-Pound	t_R_ (*E*)-Isomer	Area Ratio ^4^ A_315_/_0_	t_R_ (*Z*)-Isomer	Area (Z)-Isomer	t_R_ NAC-1	Area NAC-1	t_R_ NAC-2	Area NAC-2
**3.2**	**2b**	16.8	0.69	ND ^5^	ND ^5^	15.8	738	16.2	412
**3.2**	**2c**	16.4	0.76	ND ^5^	ND ^5^	15.2	660	ND ^5^	-
**6.3**	**2b**	17.2	0.92	17.0	119	16.2	1607	16.3	327
**6.3**	**2c**	16.8	0.94	ND ^5^	ND ^5^	15.7	4050	ND ^5^	
**8.0**	**2b**	16.9	0.68	ND ^5^	ND ^5^	15.9	6372	16.0	3545
**8.0**	**2c**	16.8	0.67	ND ^5^	ND ^5^	15.7	7564	15.8	4785

^1^ Retention times in minutes; ^2^ Data refer to the average of two independent measurements at the 315 min timepoint; ^3^ pH value of the aqueous thiol solution; ^4^ Ratios of peak areas of the starting compounds (**2b**, **2c**) measured at 0 and 315 min; ^5^ Not detectable.

**Table 4:** Reactivity indices were obtained for **1a** [1], **1b, 1c, 2a, 2b, 2c, 3a** [1], **3b, 3c CH_3_SH** [1] and **CH_3_S^−^** [1] at the M06-2X/6-311++G(d,p) level of theory.

Descriptors	1a [1] kcal.mol^−1^	1b kcal.mol^−1^	1c kcal.mol^−1^	2a kcal.mol^−1^	2b kcal.mol^−1^	2c kcal.mol^−1^	3a [1] kcal.mol^−1^	3b kcal.mol^−1^	3c kcal.mol^−1^	CH_3_SH kcal.mol^−1^	CH_3_S^-^ kcal.mol^−1^
E_HOMO_	−183.24	−177.98	−170.84	−181.22	−176.17	−169.03	−179.86	−175.34	−168.25	−184.76	−173.50
E_LUMO_	−35.98	−34.03	−31.96	−31.57	−30.30	−29.08	−30.36	−27.09	−25.65	−2.98	78.00
ΔE_HOMO-LUMO_	147.27	143.95	138.89	149.64	145.87	139.94	149.50	148.25	142.60	181.78	251.50
Chemical potential (μ)	−109.61	−106.01	−101.40	−106.40	−103.24	−99.06	−105.11	−101.22	−96.95	−93.87	47.28
Chemical hardness (*η*)	147.27	143.95	138.89	149.64	145.87	139.94	149.50	148.25	142.60	181.78	251.50
Electrophylicity index (*ω*)	40.79	39.03	37.02	37.82	36.53	35.06	36.95	34.55	32.96	−24.24	4.82

## Data Availability

The original contributions presented in the study are included in the article/Supplementary Materials, further inquiries can be directed to the corresponding author/s.

## Supplementary Materials

The following supporting information (Figures S1–S30) can be downloaded at https://www.mdpi.com/article/10.3390/ijms25147773/s1.
