# Supplementary material for: (E)-2-Benzylidenecyclanones: Part XIX. Reaction of (E)-2-(4′-X-Benzylidene)-1-tetralones with Cellular Thiols: Comparison of Thiol Reactivities of Open-Chain Chalcones and Their Six- and Seven-Membered Cyclic Analogs
Source: Int J Mol Sci. 2024 Jul 16;25(14):7773. doi: 10.3390/ijms25147773 (PMC11277078; doi:10.3390/ijms25147773)
Supplement: Supplementary file 1 [file ijms-25-07773-s001.zip › ijms-3010594-supplementary.pdf]

Supplementary

(E)-2-Benzylidenecyclanones: Part XIX. Reaction of (E)-2-(4′-X-Benzylidene)-1-Tetralones with Cellular Thiols: Comparison of Thiol Reactivities of Open-Chain Chalcones and Their Six- and Seven-Membered Cyclic Analogs

Fatemeh Kenari[1], Zoltán Pintér[1], Szilárd Molnár[1,2], Igor D. Borges[3], Ademir J. Camargo[3], Hamilton B. Napolitano[3] and Pál Perjési[1,3] *

1 Institute of Pharmaceutical Chemistry, University of Pécs, H-7624 Pécs, Hungary; kenari.fatemeh@gmail.com (F.K.); pinter.zoltan@pte.hu (Z.P.); molnar.szilard@pte.hu (S.M.)
2 Research Institute for Viticulture and Oenology, University of Pécs, H-7634 Pécs, Hungary
3 Grupo de Química Teórica e Estrutural de Anápolis, Universidade Estadual de Goiás, Anápolis 75132-903, GO, Brazil; i.dalarmelino@gmail.com (I.D.B.); ajc@ueg.br (A.J.C.); hamilton@ueg.br (H.B.N.)
* Correspondence: pal.perjesi@gytk.pte.hu; Tel.: +36-72-503-650

Abstract: Non-enzyme-catalyzed thiol addition onto the α,β-unsaturated carbonyl system is associated with several biological effects. Kinetics and diastereoselectivity of non-enzyme catalyzed nucleophilic addition of GSH and NAC to the six-membered cyclic chalcone analogs **2a** and **2b** were investigated at different pH values (pH 3.2, 7.4, and 8.0). The selected compounds displayed *in vitro* cancer cell cytotoxicity (IC_{50}) of different orders of magnitude. The chalcones intrinsically reacted with both thiols under all incubation conditions. The initial rates and compositions of the final mixtures depended both on the substitution and the pH. The stereochemical outcome of the reactions was evaluated by HPLC-UV method. The structure of the formed thiol-conjugates and the retro-Michael products (*Z*)-**2a** and (*Z*)-**2b** were confirmed by HPLC-MS. The frontier molecular orbitals and the Fukui function calculations were carried out to investigate their effects on the six-membered cyclic analogs. Data were compared with those obtained with the open-chain (**1**) and the seven-membered (**3**) analogs. The observed reactivities do not directly relate to the difference in *in vitro* cancer cell cytotoxicity of the compounds.

Keywords: chalcone; glutathione; cysteine; thiols; Michael addition; diastereoselective addition

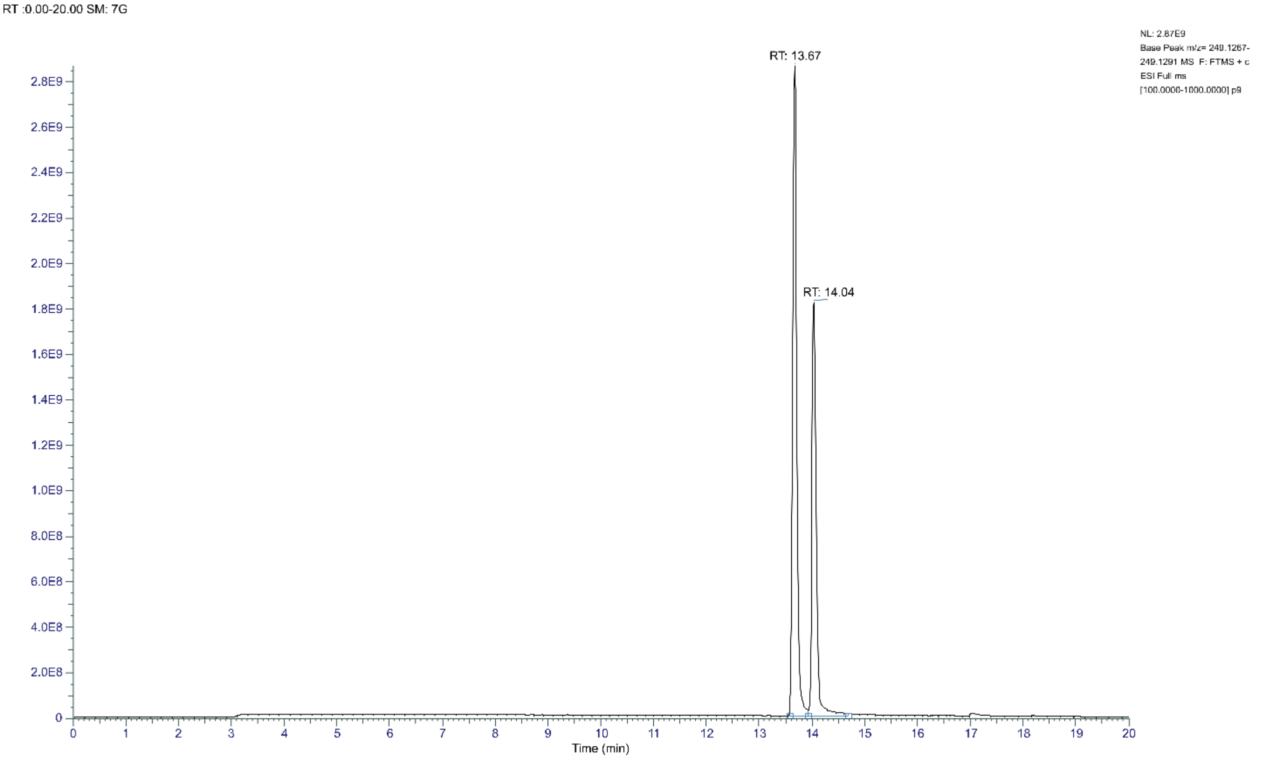

Figure S1. High resolution, positive mode HESI MS chromatogram of **2b** irradiated with natural light. (t_r 13.67 min: (*Z*)-**2b**. t_r 14.04 min: **2b**. (Extracted ion chromatogram of m/z 249.1279 [(**2b**)+H]$^+$).

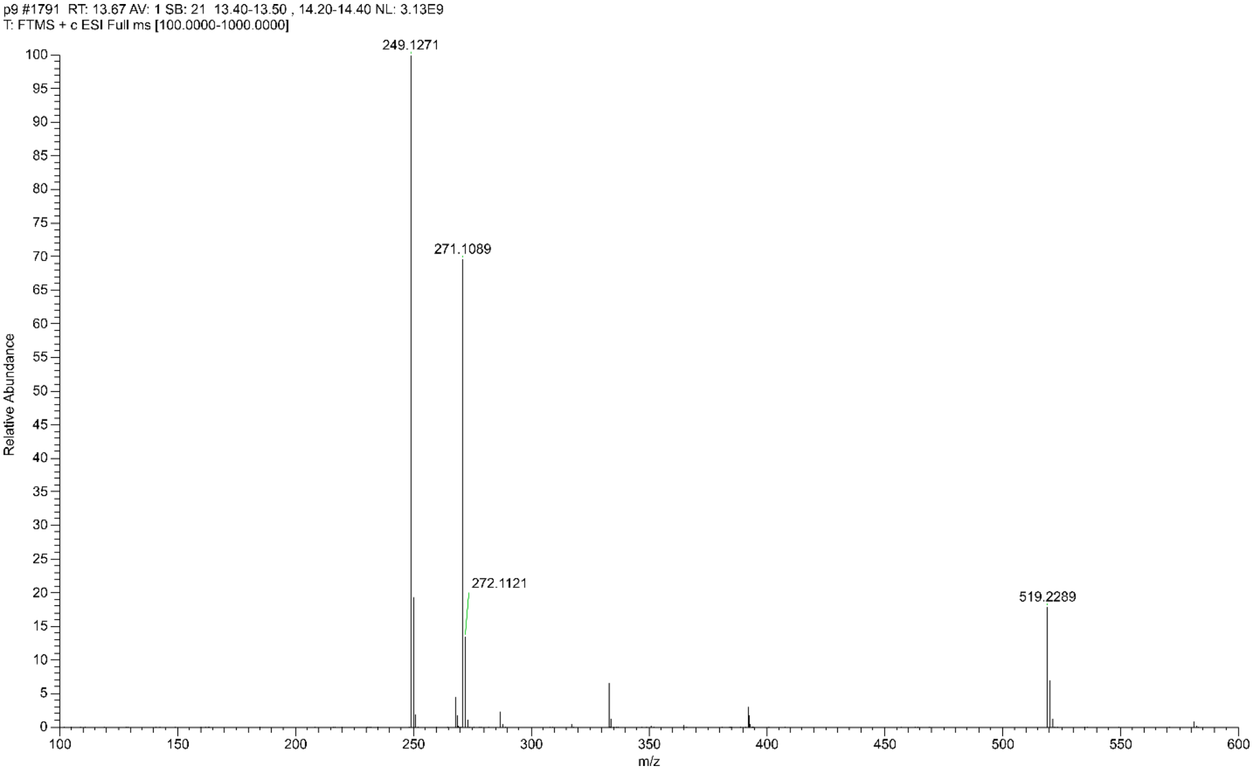

Figure S2. High resolution, positive mode HESI MS spectrum of (*Z*)-**2b.** (m/z 249.1271 [(**2b**)+H]$^+$, m/z 271.1089 [(**2b**)+Na]$^+$ and m/z 519.2289 [(**2b**)$_2$+Na]$^+$).

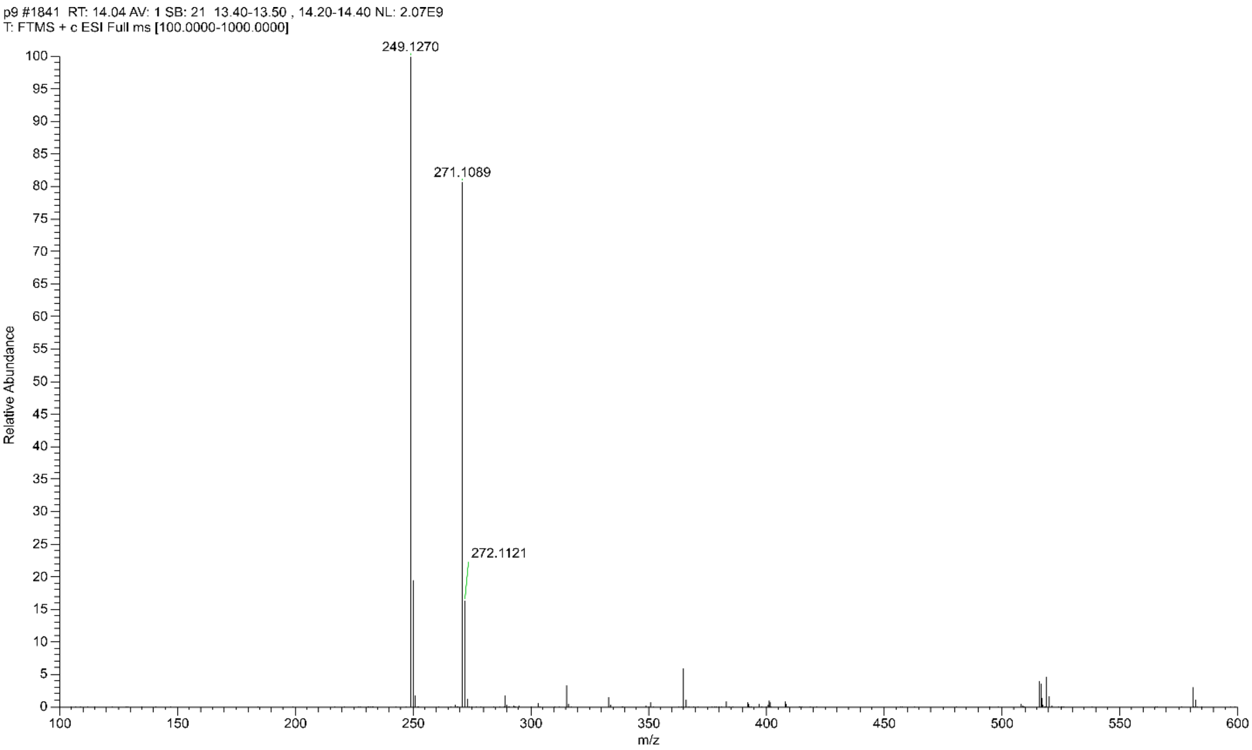

Figure S3. High resolution, positive mode HESI MS spectrum of **2b**. (m/z 249.1270 [(**2b**)+H]$^+$ and m/z 271.1089 [(**2b**)+Na]$^+$).

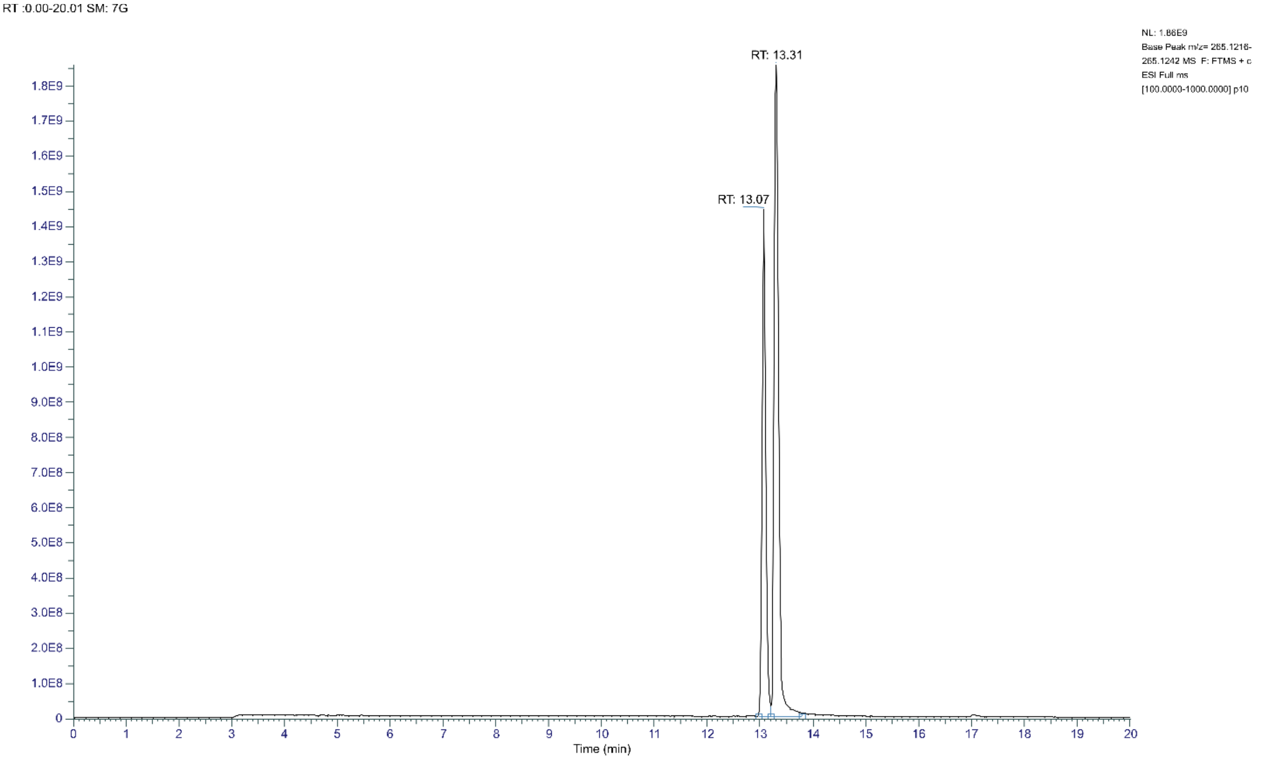

Figure S4. High resolution, positive mode HESI MS chromatogram of **2c** irradiated with natural light. (t_r 13.07 min: (*Z*)-**2c**. t_r 13.31 min: **2c**. (Extracted ion chromatogram of m/z 265.1229 [(**2c**)+H]$^+$).

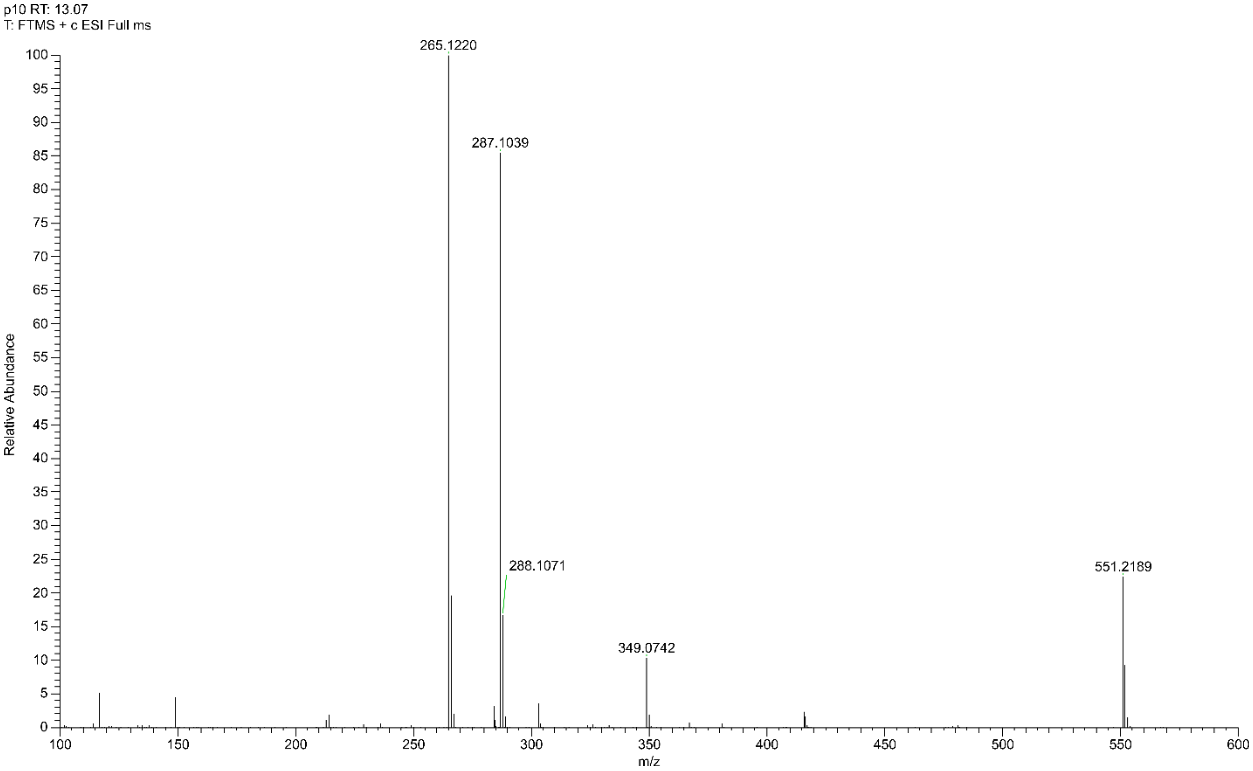

Figure S5. High resolution, positive mode HESI MS spectrum of (*Z*)-**2c**. (m/z 265.1220 [(**2c**)+H]$^+$, m/z 287.1039 [(**2c**)+Na]$^+$-adduct and m/z 551.2189 [(**2c**)$_2$+Na]$^+$-adduct).

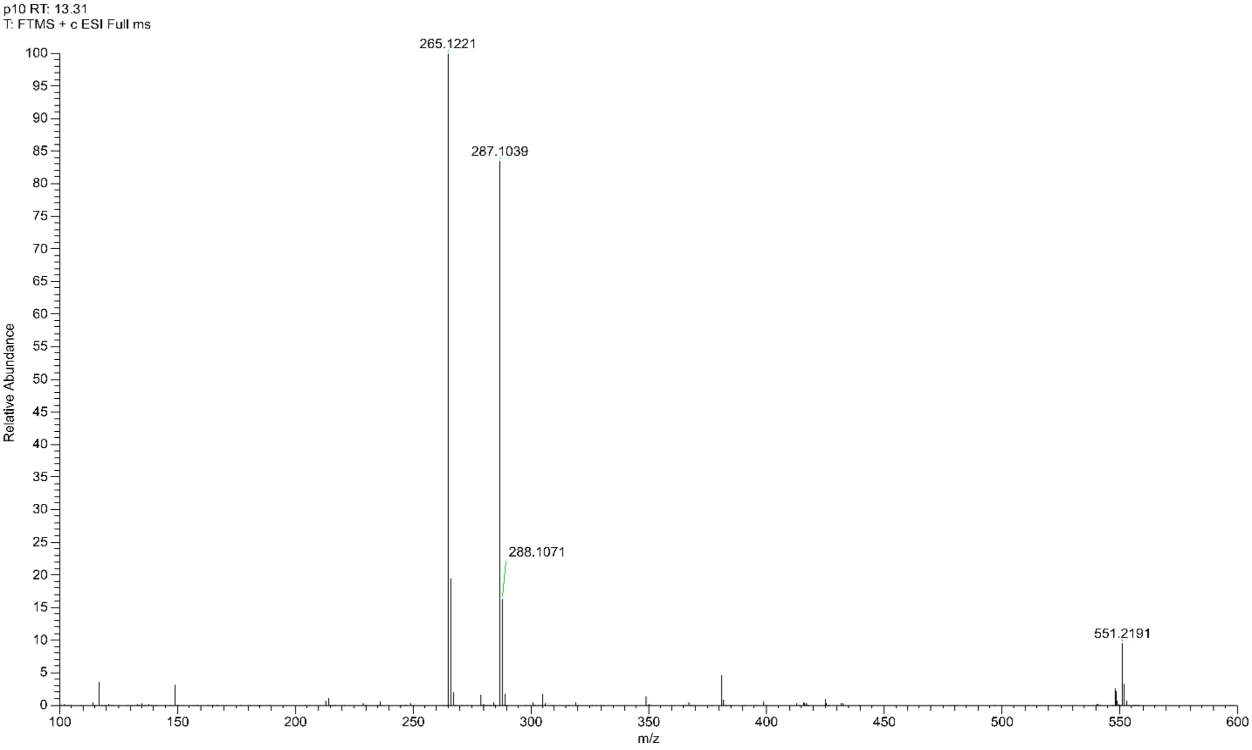

Figure S6. High resolution, positive mode HESI MS spectrum of **2c**. (m/z 265.1221 [(**2c**)+H]$^+$, m/z 287.1039 [(**2c**)+Na]$^+$-adduct and m/z 551.2191 [(**2c**)$_2$+Na]$^+$-adduct).

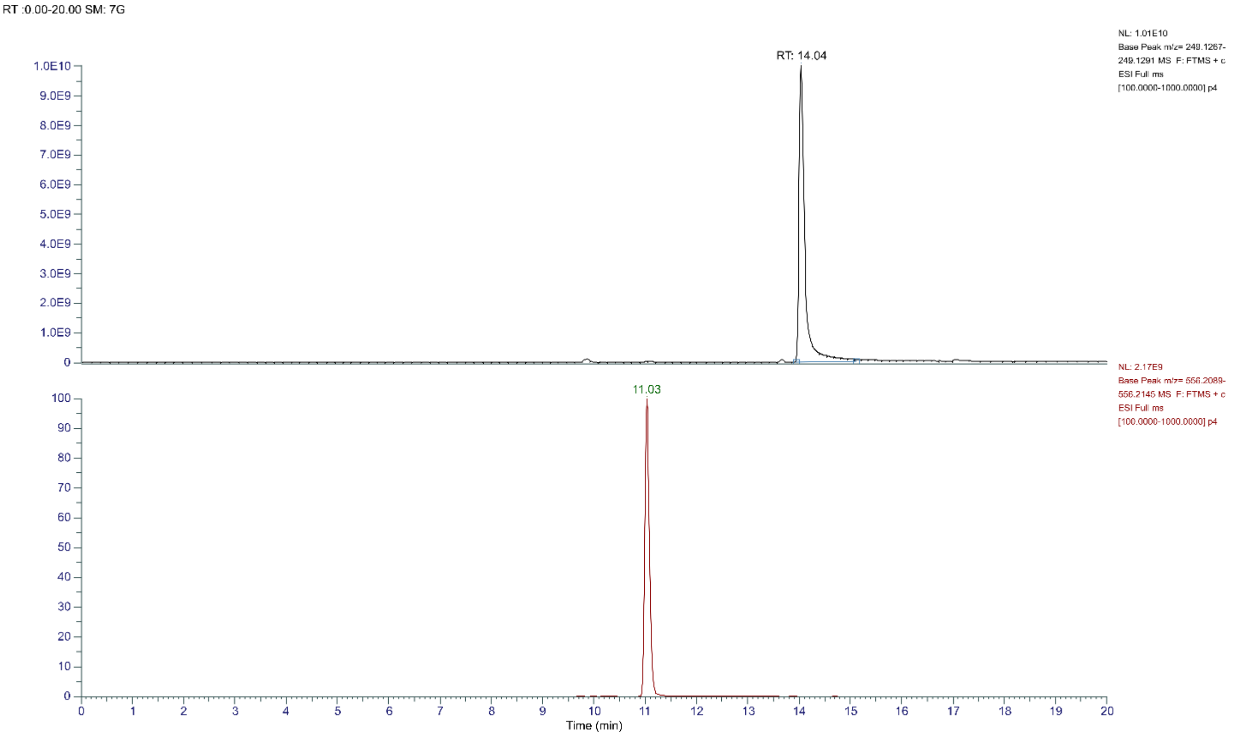

Figure S7. High resolution, positive mode HESI MS chromatograms of the **2b**/GSH incubate (pH 3.2; 315-minute sample). Upper panel: m/z 249.1279 [(**2b**)+H]$^+$ t_r 14.04 min: **2b**. Lower panel: m/z 556.2117 [(**2b-GSH**)+H]$^+$ t_r 11.03 min: **2b-GSH**.

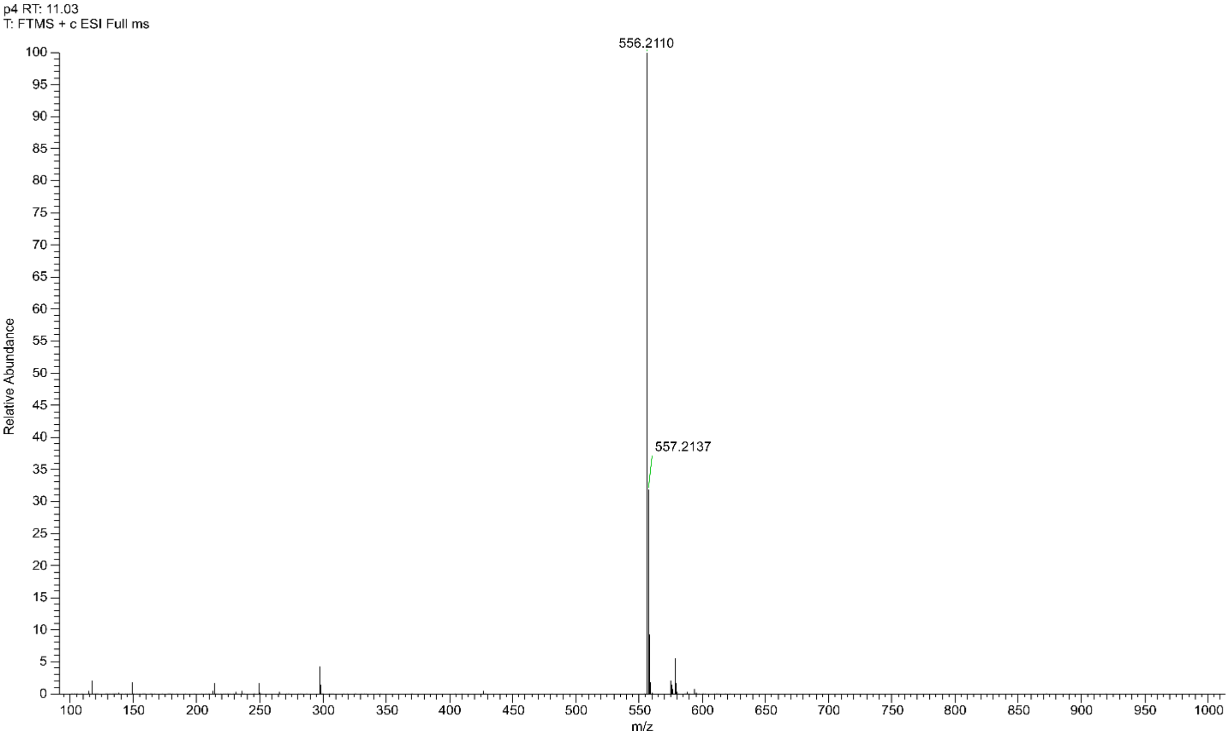

Figure S8. High resolution, positive mode HESI MS spectrum of the **2b-GSH** conjugate (t_r 11.03 min) formed in the 315-minute sample of the pH 3.2 incubate. (m/z 556.2110 [(**2b-GSH**)+H]$^+$).

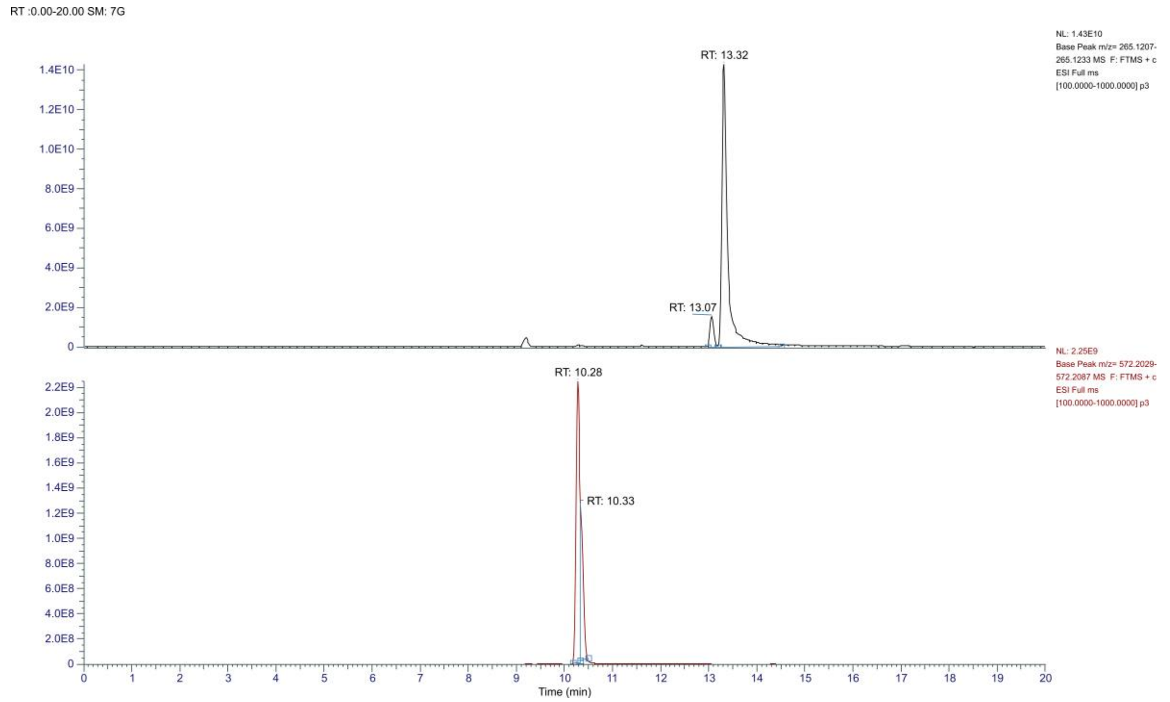

Figure S9. High resolution, positive mode HESI MS chromatograms of the **2c**/GSH incubate (pH 3.2; 315-minute sample). Upper panel: m/z 265.1229 [(**2c**)+H]$^+$ t_r 13.32 min: **2c**. Lower panel: m/z 572.2067 [(**2c-GSH**)+H]$^+$ t_r 10.28 min: **2c-GSH-1**, t_r 10.33 min: **2c-GSH-2**.

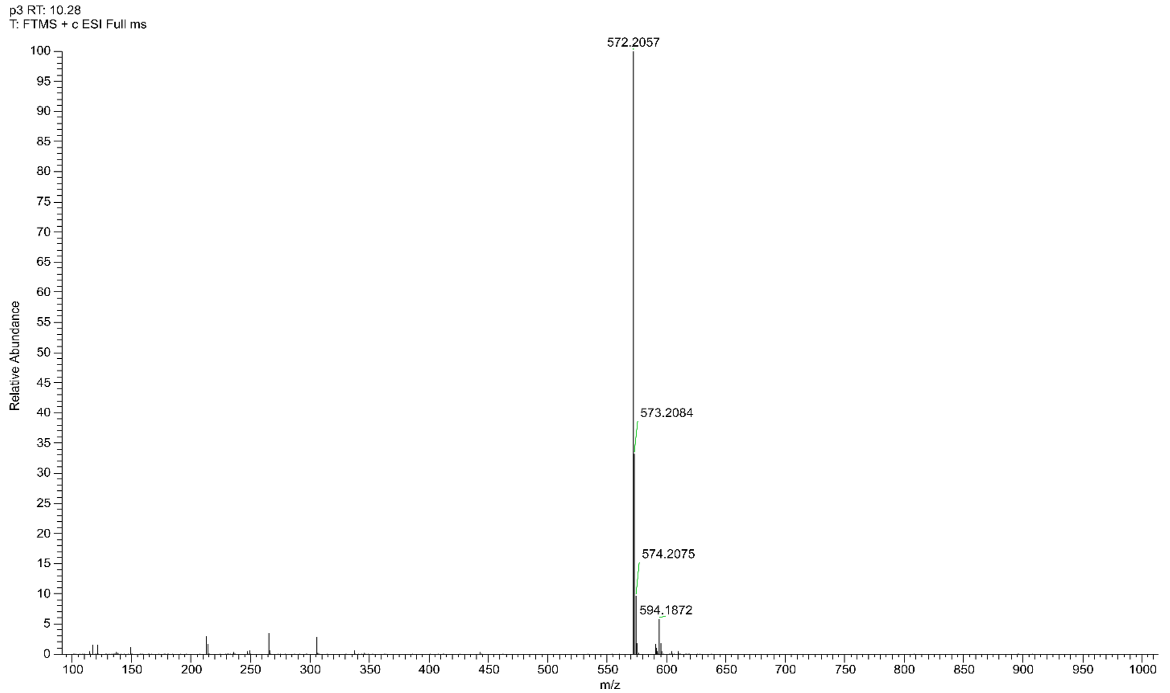

Figure S10. High resolution, positive mode HESI MS spectrum of the **2c-GSH-1** conjugate (t_r 10.28 min) formed in the 315-minute sample of the pH 3.2 incubate. (m/z 572.2057 [(**2c-GSH**)+H]$^+$).

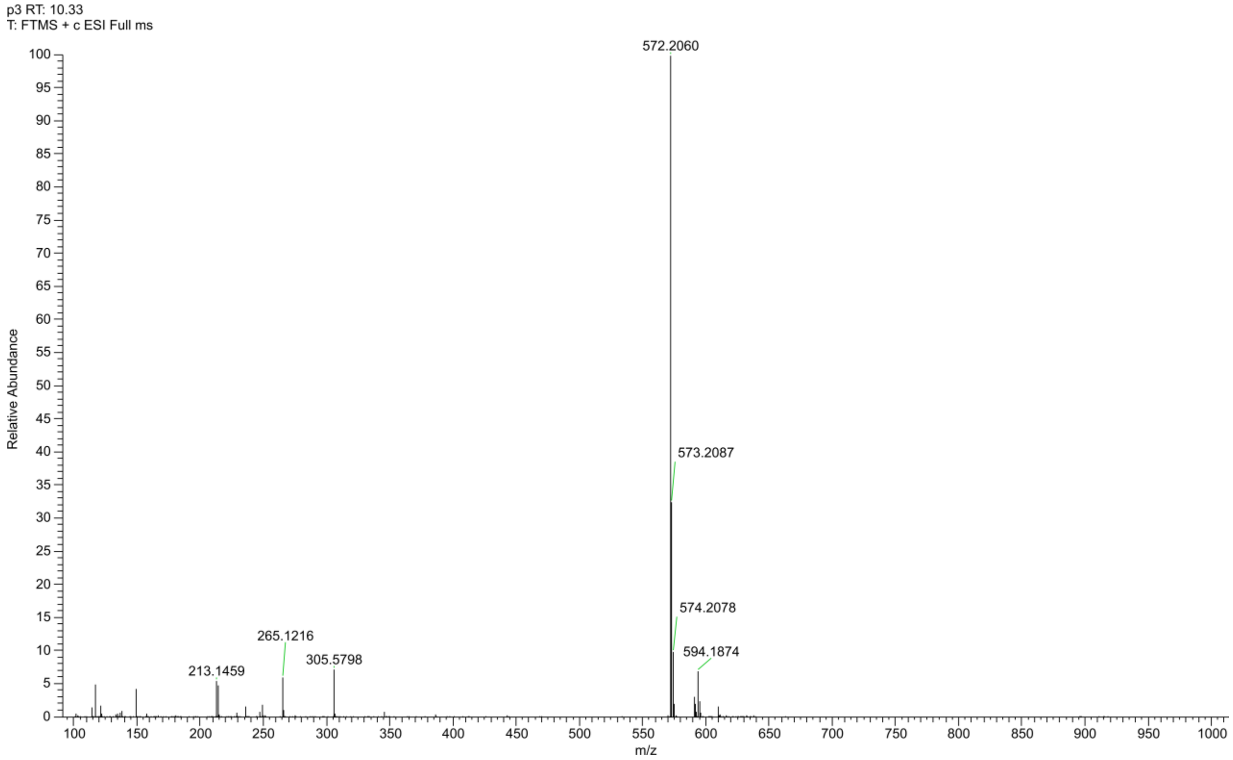

Figure S11. High resolution, positive mode HESI MS spectrum of the **2c-GSH-2** conjugate (t_r 10.33 min) formed in the 315-minute sample of the pH 3.2 incubate. (m/z 572.2060 [(**2c-GSH**)+H]$^+$).

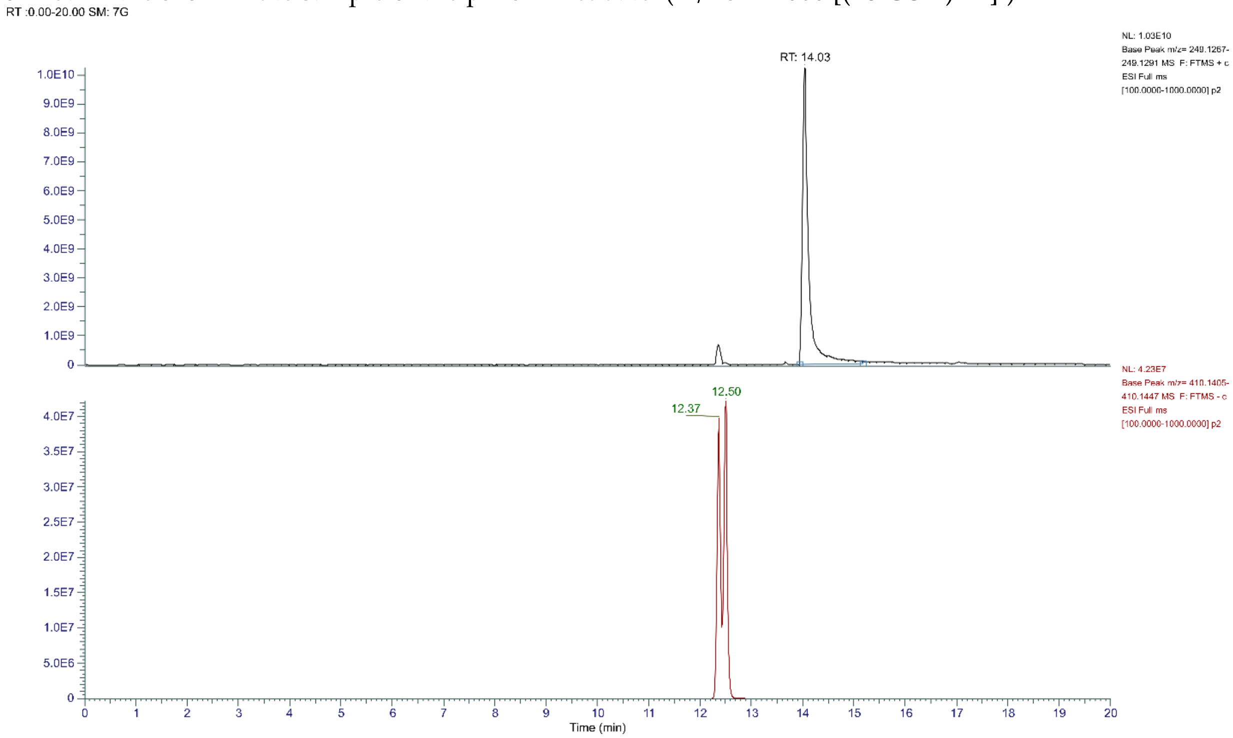

Figure S12. High resolution, HESI MS chromatograms of **2b**/NAC incubate (pH 3.2; 315-minute sample). Upper panel: positive mode m/z 249.1279 [**2b**+H]$^+$ t_r 14.03 min: **2b**. Lower panel: negative mode m/z 410.1426 [(**2b-NAC**)-H]$^-$ t_r 12.37 min: **2b-NAC-1**, t_r 12.50 min: **2b-NAC-2.**

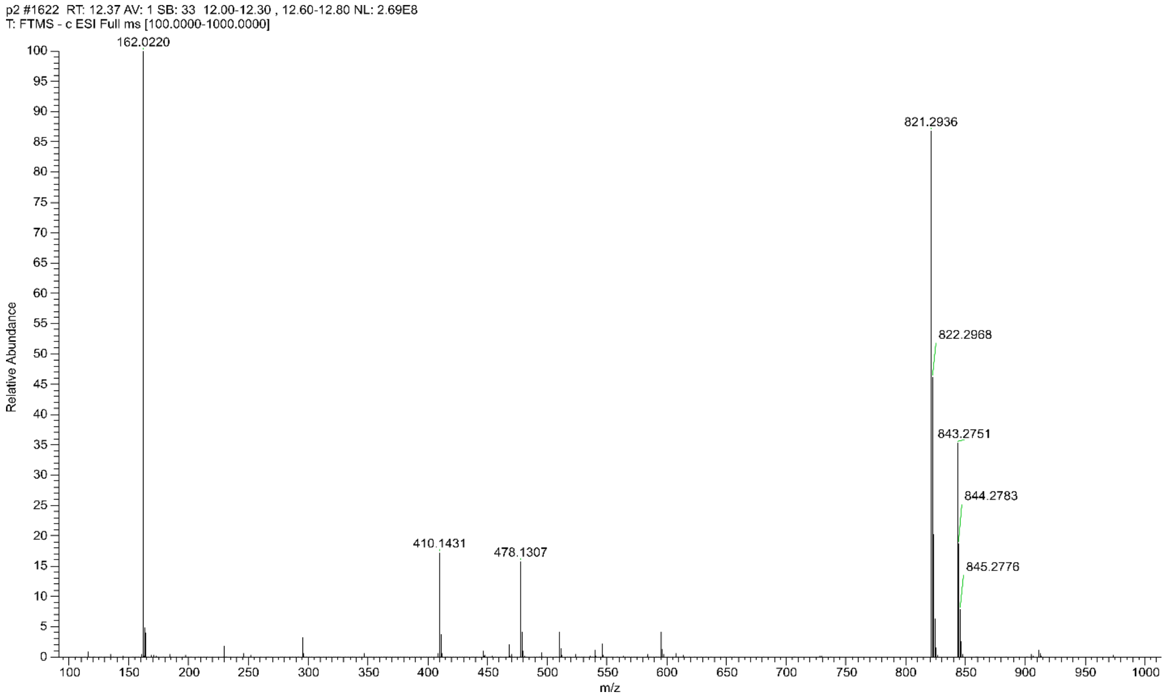

Figure S13. High resolution, negative mode HESI MS spectrum of the **2b-NAC-1** conjugate (t_r 12.37 min) formed in the 315-minute sample of the pH 3.2 incubate. (m/z 410.1431 [(**2b-NAC**)-H]$^-$, m/z 478.1307 [(**2b-NAC**)+Na-formate clusters-H]$^-$, m/z 821.2936 [(**2b-NAC**)$_2$-H]$^-$, m/z 843.2751 [(**2b-NAC**)$_2$+Na-2H]$^-$).

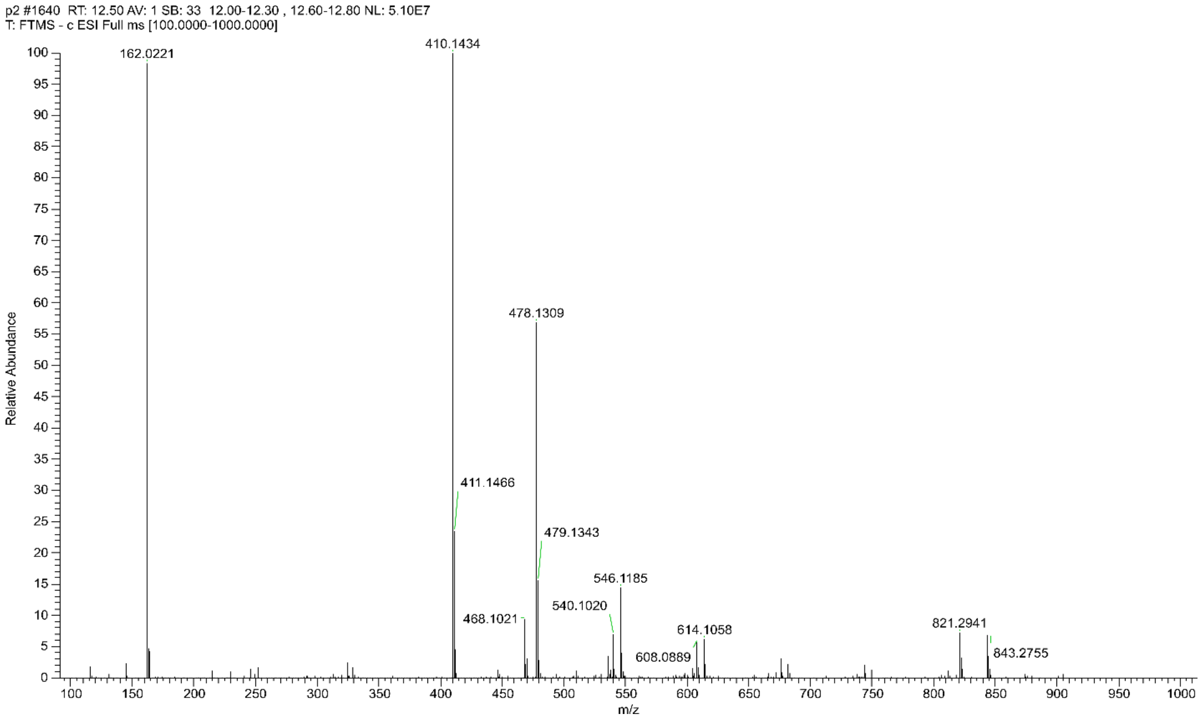

Figure S14. High resolution, negative mode HESI MS spectrum of the **2b-NAC-2** conjugate (t_r 12.50 min) formed in the 315-minute sample of the pH 3.2 incubate. (m/z 410.1434 [(**2b-NAC**)-H]$^-$, m/z 478.1309 [(**2b-NAC**)+Na-formate clusters-H]$^-$, m/z 821.2941 [(**2b-NAC**)$_2$-H]$^-$, m/z 843.2755 [(**2b-NAC**)$_2$+Na-2H]$^-$).

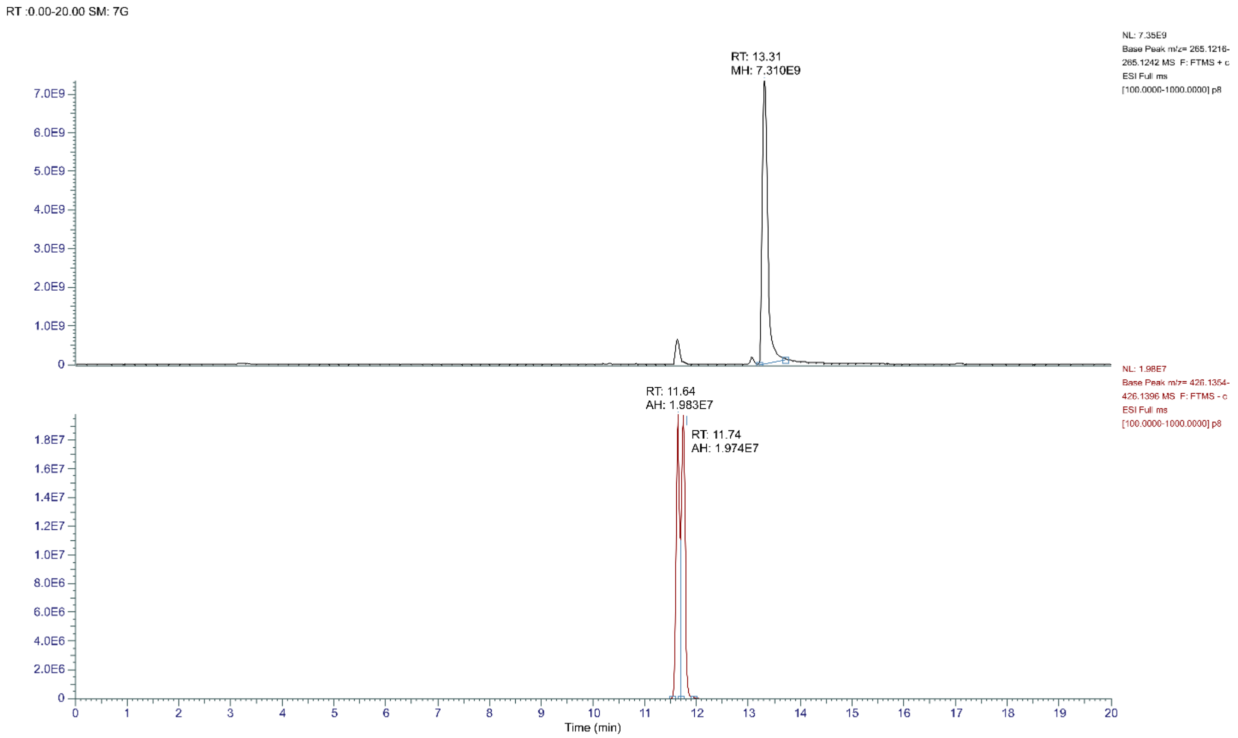

Figure S15. High resolution, HESI MS chromatograms of the **2c**/NAC incubate (pH 3.2, 315-minute sample). Upper panel: positive mode m/z 265.1229 [(**2c**)+H]$^+$ t_r 14.03 min: **2c**. Lower panel: negative mode m/z 426.1375 [(**2c**)-H]$^-$ t_r 11.64 min: **2c-NAC-1**, t_r 11.74 min: **2c-NAC-2**.

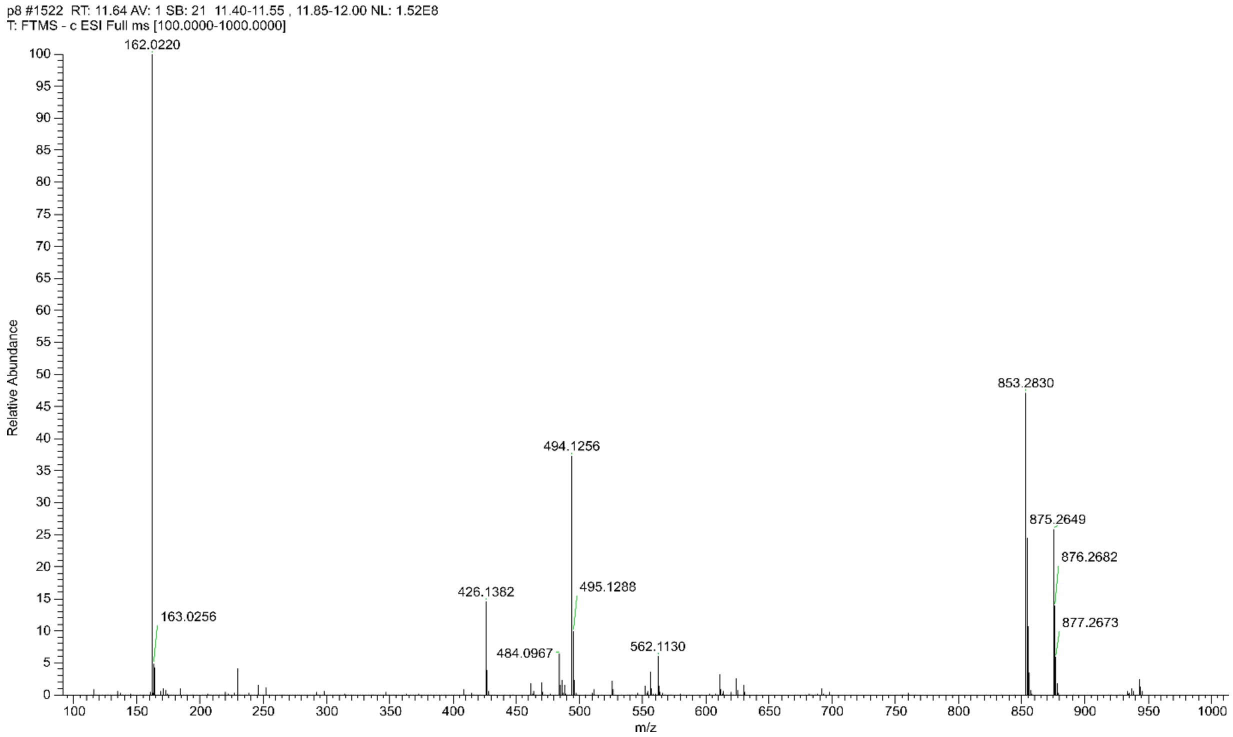

Figure S16. High resolution, negative mode HESI MS spectrum of the **2c-NAC-1** conjugate (t_r 11.64 min) formed in the 315-minute sample of the pH 3.2 incubate. (m/z 426.1362 [(**2c-NAC**)-H]$^-$, m/z 494.1249 [(**2c-NAC**)+Na-formate clusters-H]$^-$, m/z 853.2830 [(**2c-NAC**)$_2$-H]$^-$, m/z 875.2649 [(**2c-NAC**)$_2$+Na-2H]$^-$).

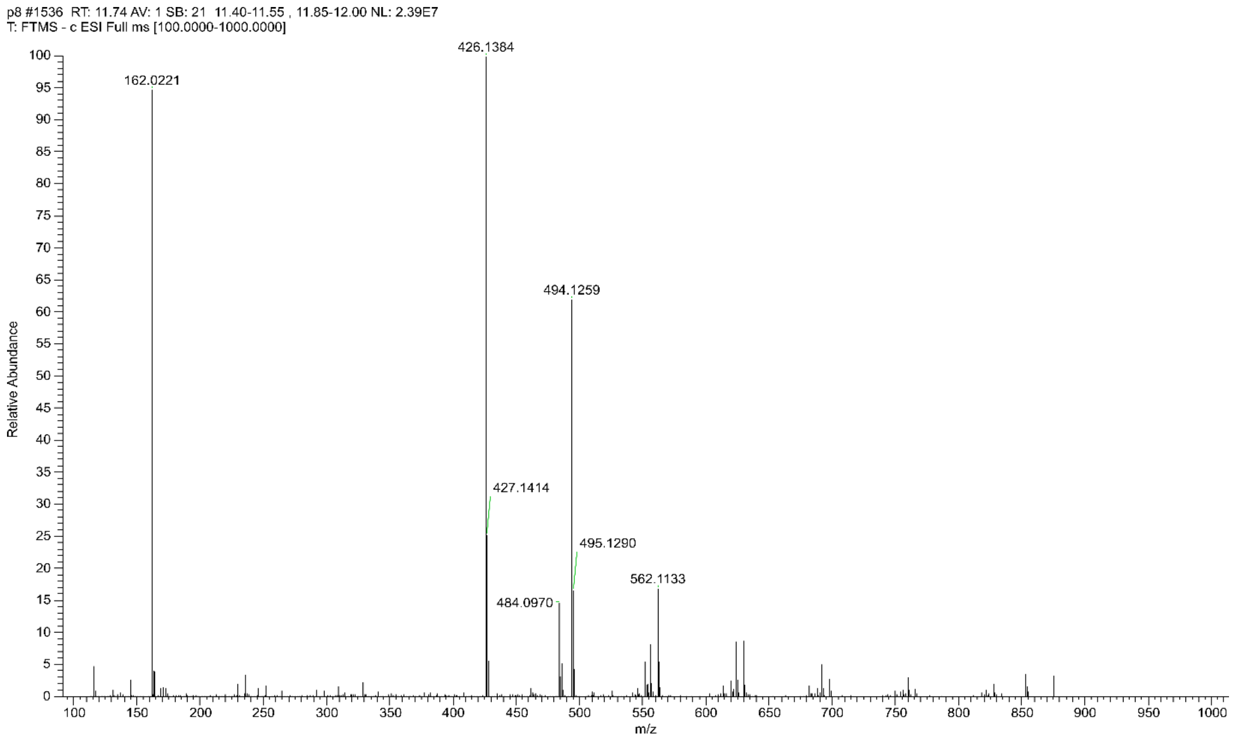

Figure S17. High resolution, negative mode HESI MS spectrum of the **2c-NAC-2** conjugate (t_r 11.74 min) formed in the 315-minute sample of the pH 3.2 incubate. (m/z 426.1364 [(**2c-NAC**)-H]$^-$, m/z 494.1259 [(**2c-NAC**)+Na-formate clusters-H]$^-$).

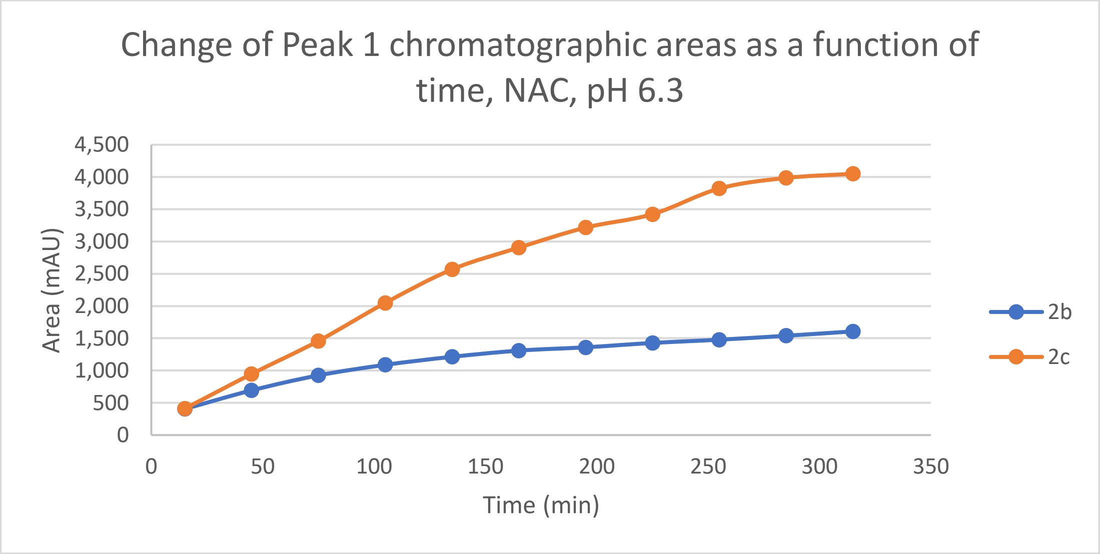

Figure S18. Change in the chromatographic peak area of adduct 1 of **2b** and **2c** in the chalcone-NAC incubations at pH 6.3.

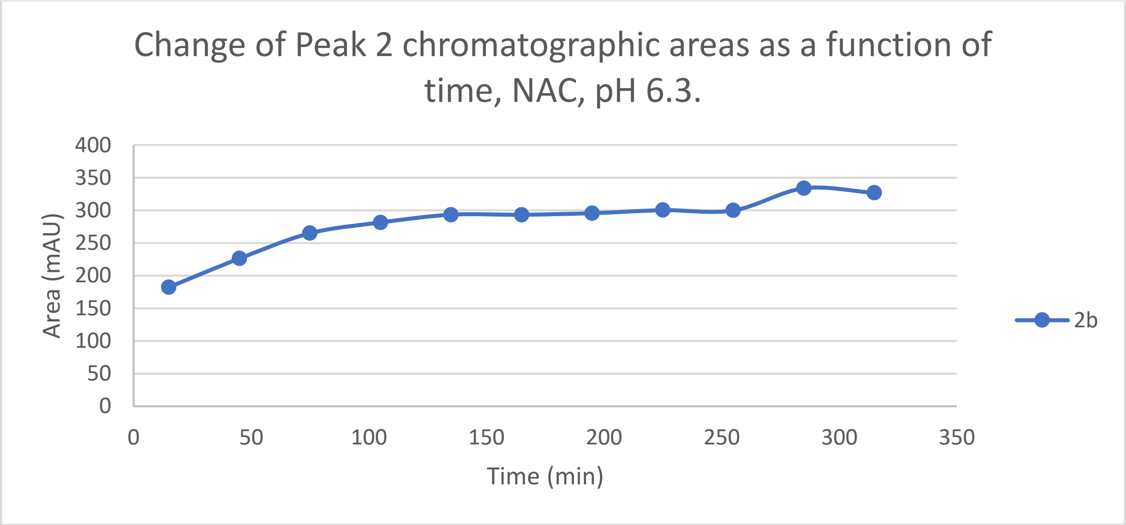

Figure S19. Change in the chromatographic peak area of adduct 2 of **2b** in the chalcone-NAC incubations at pH 6.3.

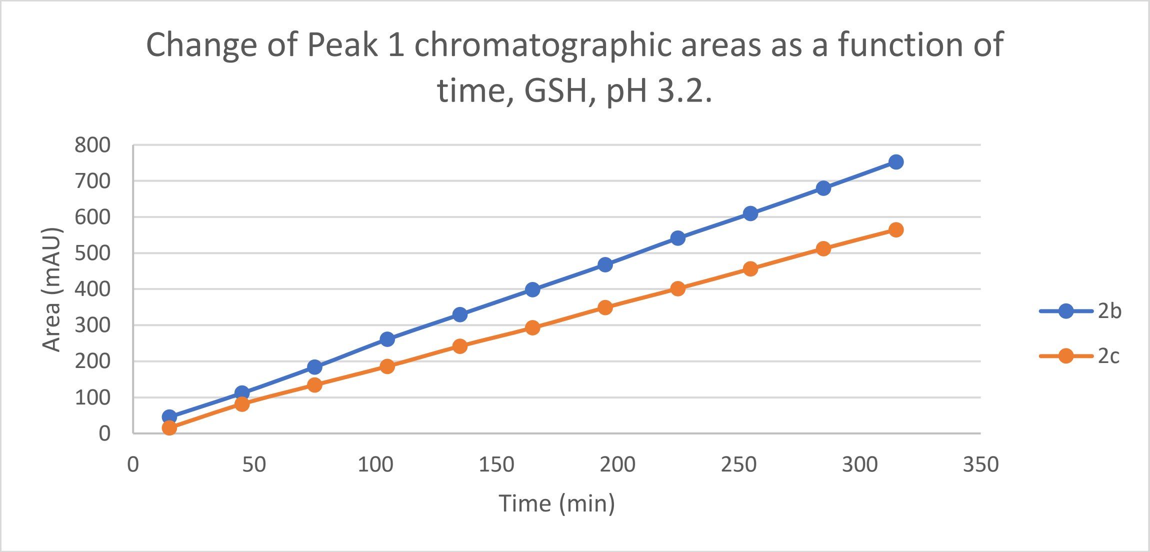

Figure S20. Change in the chromatographic peak area of adduct 1 of **2b** and **2c** in the chalcone-GSH incubations at pH 3.2.

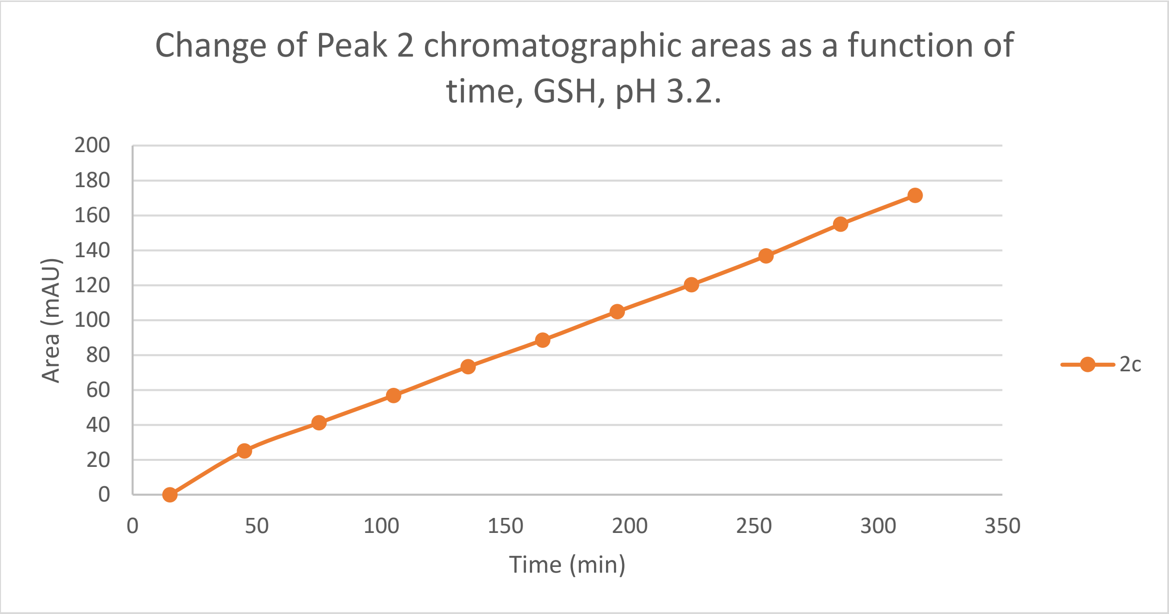

Figure S21. Change in the chromatographic peak area of adduct 2 of **2c** in the chalcone-NAC incubations at pH 3.2.

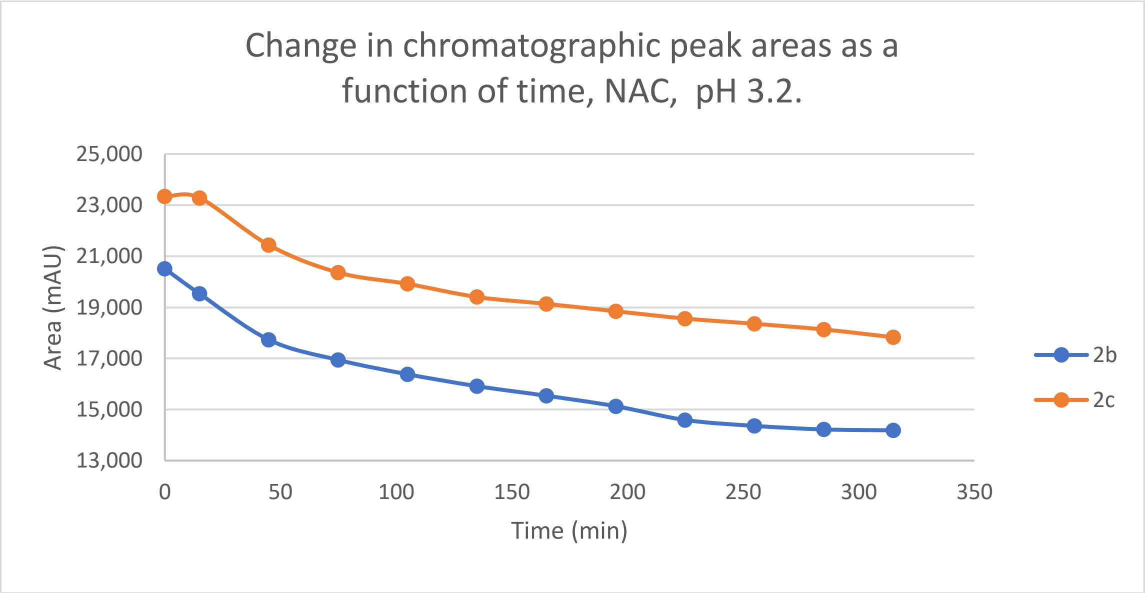

Figure S22. Change in the chromatographic peak area of chalcones **2b** and **2c** in the chalcone–NAC incubations at pH 3.2.

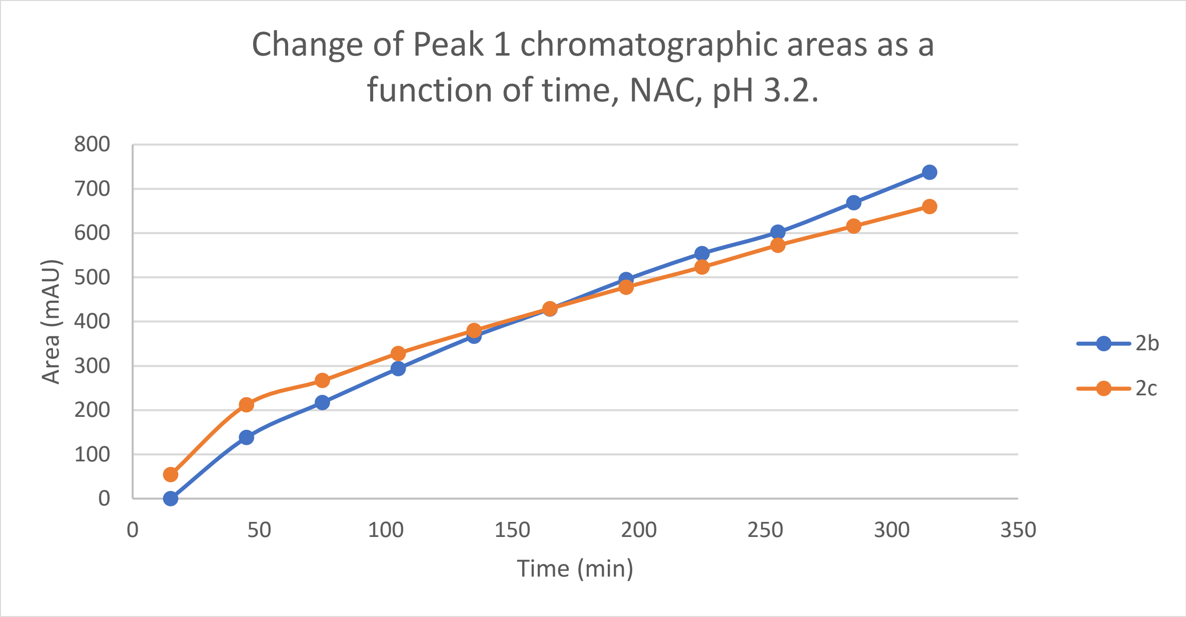

Figure S23. Change in the chromatographic peak area of adduct 1 of **2b** and **2c** in the chalcone-NAC incubations at pH 3.2.

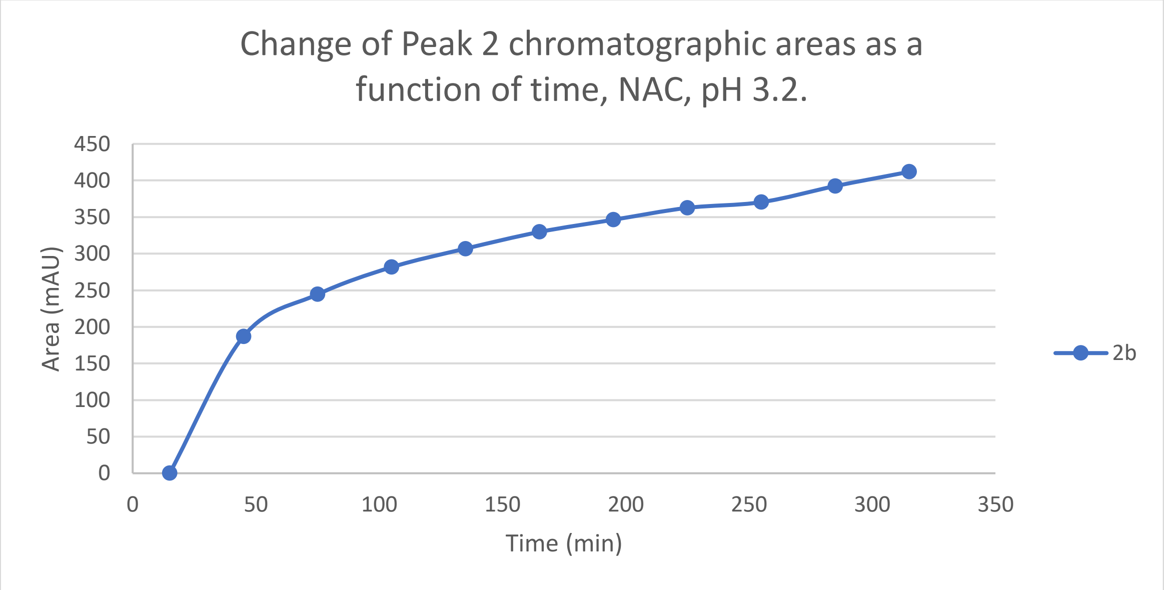

Figure S24. Change in the chromatographic peak area of adduct 2 of **2b** in the chalcone-NAC incubations at pH 3.2.

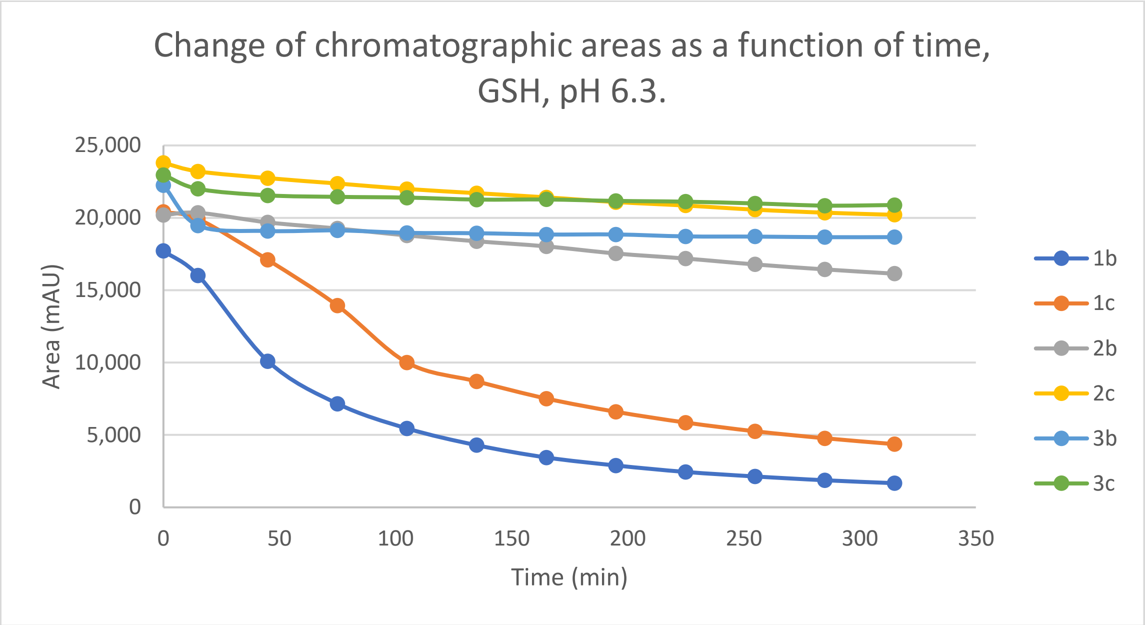

Figure S25. Change in the chromatographic peak area of chalcones **1b, 1c** [25], **2b**, **2c,** and **3b, 3c** [1] in the chalcone-GSH incubations at pH 6.3.

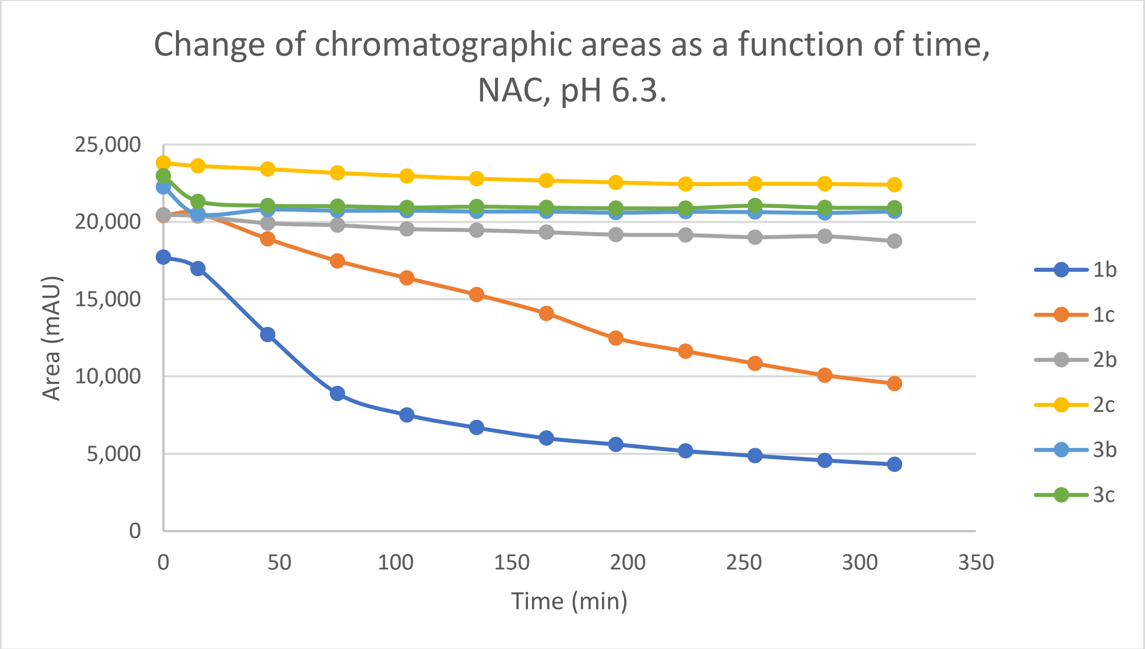

Figure S26. Change in the chromatographic peak area of chalcones **1b, 1c** [25]**, 2b**, **2c,** and **3b, 3c** [1] in the chalcone-NAC incubations at pH 6.3.

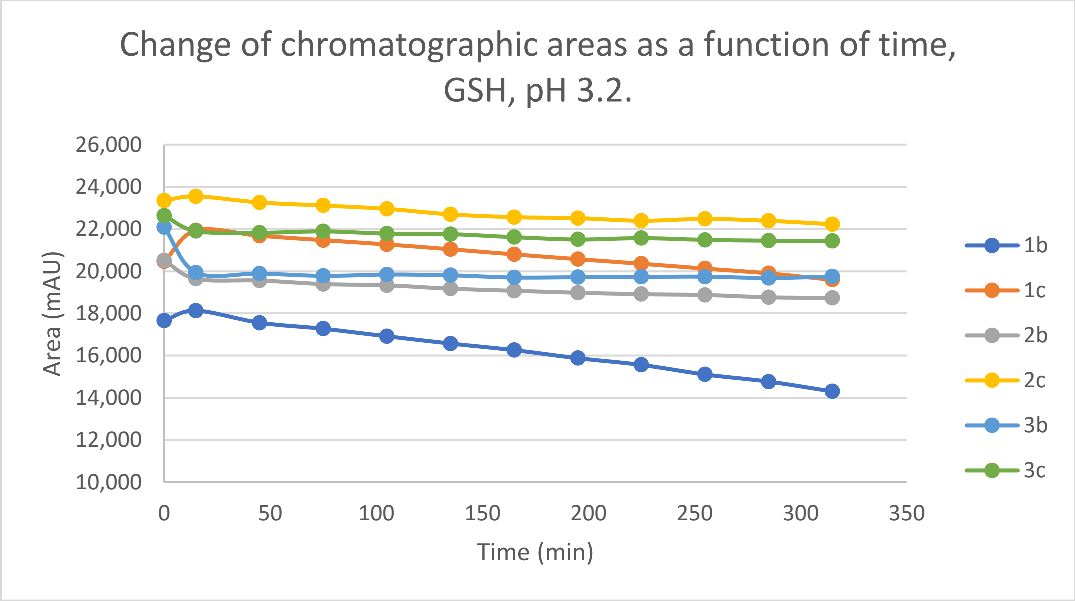

Figure S27. Change in the chromatographic peak area of chalcones **1b, 1c** [25]**, 2b**, **2c, and 3b, 3c** [1] in the chalcone-GSH incubations at pH 3.2.

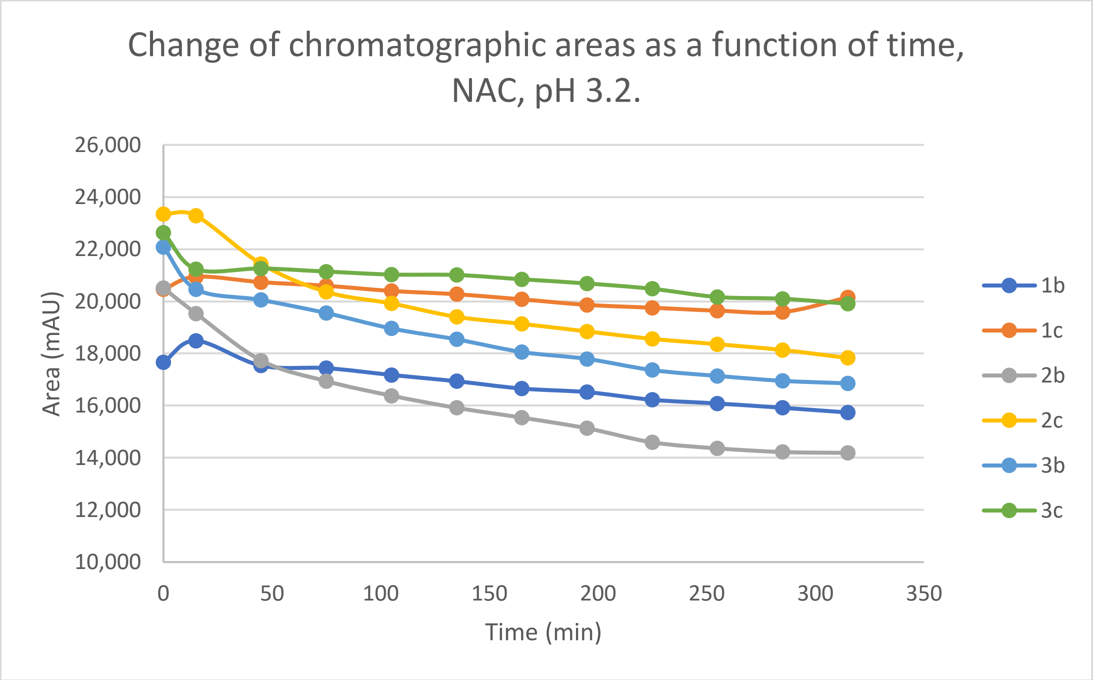

Figure S28. Change in the chromatographic peak area of chalcones **1b, 1c** [25]**, 2b**, **2c, and 3b, 3c [**1] in the chalcone-NAC incubations at pH 3.2.

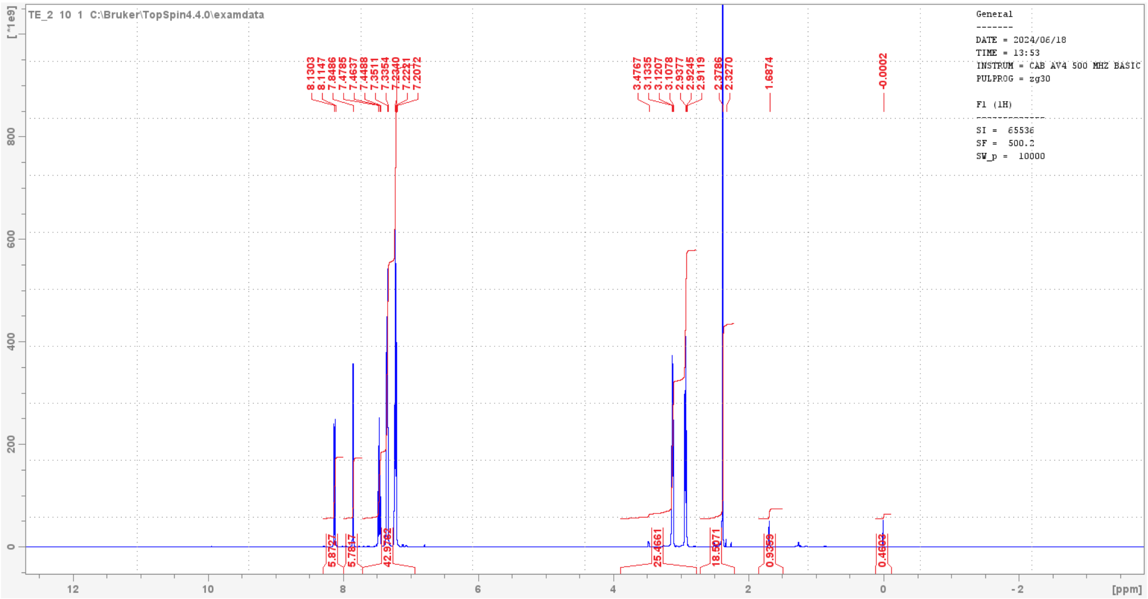

Figure S29. ^{1}H NMR spectrum (in $CDCl_3$) of **2b**.

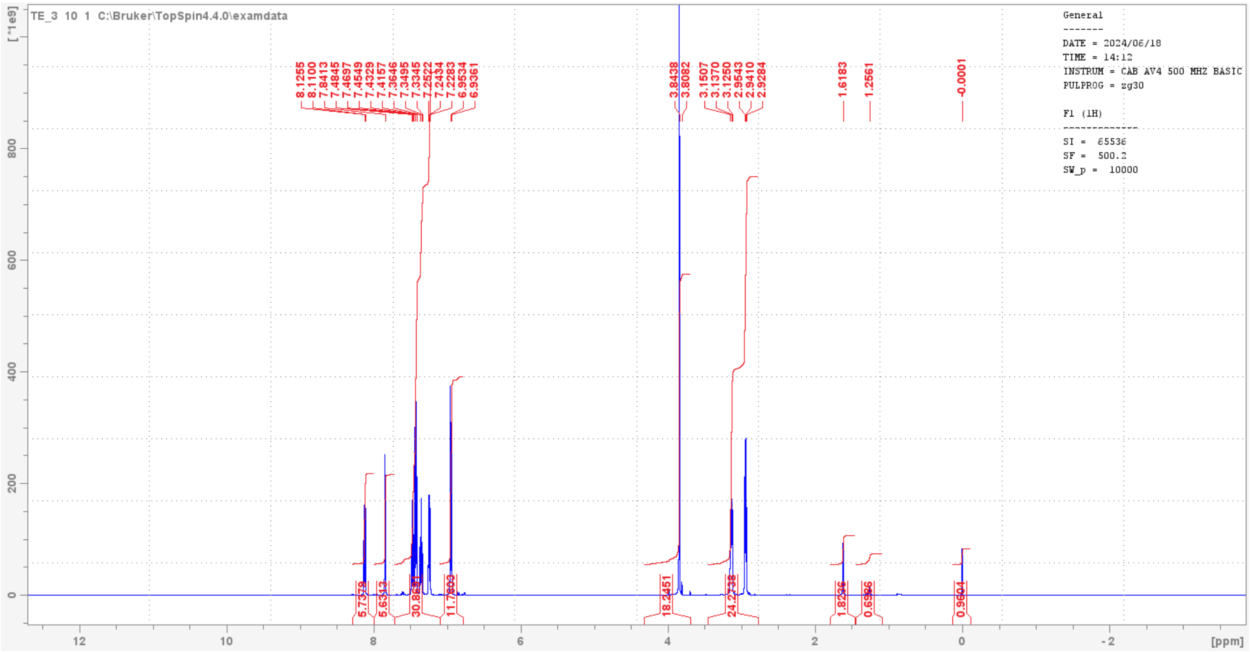

Figure S30. ^{1}H NMR spectrum (in $CDCl_3$) of **2b**.
